# Serum FGF21 as a predictor of response to atezolizumab and bevacizumab in HCC

**DOI:** 10.1016/j.jhepr.2025.101364

**Published:** 2025-02-19

**Authors:** Risako Kohya, Goki Suda, Masatsugu Ohara, Shunichi Hosoda, Takuya Sho, Makoto Chuma, Atsumasa Komori, Yuki Kugiyama, Yutaka Yasui, Kaoru Tsuchiya, Masayuki Kurosaki, Joji Tani, Shun Kaneko, Mina Nakagawa, Yasuhiro Asahina, Shinya Maekawa, Nobuyuki Enomoto, Yoshiya Yamamoto, Masaru Baba, Ren Yamada, Takashi Sasaki, Tomoka Yoda, Sonoe Yoshida, Qingjie Fu, Zijian Yang, Osamu Maehara, Shunsuke Ohnishi, Yoshimasa Tokuchi, Takashi Kitagataya, Naoki Kawagishi, Masato Nakai, Mitsuteru Natsuizaka, Koji Ogawa, Naoya Sakamoto

**Affiliations:** 1 Department of Gastroenterology and Hepatology, Graduate School of Medicine, Hokkaido University, Sapporo, Hokkaido, Japan; 2 Gastroenterology Centre, Yokohama City University Medical Centre, Minami-ku, Yokohama, Japan; 3 Hepatology Division, NHO Nagasaki Medical Centre, Ōmura, Nagasaki, Japan; 4 Department of Gastroenterology and Hepatology, Musashino Red Cross Hospital, Musashino, Tokyo, Japan; 5 Department of Gastroenterology and Neurology, Faculty of Medicine, Kagawa University, Takamatsu, Kagawa, Japan; 6 Department of Gastroenterology and Hepatology, Tokyo Medical and Dental University, Bunkyo-ku, Tokyo, Japan; 7 Department of Liver Disease Control, Tokyo Medical and Dental University, Bunkyo-ku, Tokyo, Japan; 8 Department of Gastroenterology and Hepatology, Faculty of Medicine, University of Yamanashi, Kofu City, Yamanashi, Japan; 9 Department of Gastroenterology and Hepatology, Hakodate Municipal Hospital, Hakodate, Hokkaido, Japan; 10 Centre for Gastroenterology and Hepatology, Japan Community Healthcare Organisation Hokkaido Hospital, Sapporo, Hokkaido, Japan; 11 Kushiro Rosai Hospital, Kushiro, Hokkaido, Japan; 12 Laboratory of Molecular and Cellular Medicine, Faculty of Pharmaceutical Sciences, Hokkaido University, Sapporo, Hokkaido, Japan

**Keywords:** FGF21, Atezolizumab, Bevacizumab, Hepatocellular Carcinoma, Immune-checkpoint inhibitor, Prognosis

## Abstract

**Background & Aims:**

Fibroblast growth factor 21 (FGF21) is a crucial regulator of cell metabolism. Tumour-secreted FGF21 has shown immune-checkpoint factor functions, and high FGF21 levels are associated with a poor prognosis for patients. However, its prognostic value and impact on treatment response in patients with hepatocellular carcinoma (HCC) treated with immune-checkpoint inhibitors (ICIs) remain unclear. Thus, this study investigated the potential of high FGF21 levels as a prognostic marker and whether traditional ICI-based therapy can improve the prognosis of patients with high FGF21 levels.

**Methods:**

In this retrospective multicentre study, patients with unresectable HCC who received atezolizumab/bevacizumab in the NORTE study group (n = 117) were classified into high (≥915 pg/ml; n = 29) and non-high (n = 88) FGF21 groups. For validation, we investigated patients treated with atezolizumab/bevacizumab in an independent cohort (n = 285). Overall survival, progression-free survival, and treatment response were compared between patients with and without high baseline FGF21 levels.

**Results:**

The median overall survival (*p* <0.001) and progression-free survival (*p* = 0.045) were significantly shorter in the high FGF21 group than in the non-high FGF21 group. Independent cohort analysis validated these results. In the overall cohort, the median progression-free survival (5.75 *vs.* 8.84 months; *p* = 0.027) and median overall survival (14.13 *vs.* 22.08 months; *p* <0.001) were significantly shorter in the high FGF21 group than in the non-high FGF21 group. The durable response (≥6 months) + complete response rate was significantly decreased in the high FGF21 group (*p* = 0.045). No patient with a high FGF21 level achieved a complete response, whereas this was achieved in 4.1% (13/319) of patients with non-high FGF21 levels. Multivariate Cox regression analysis identified high baseline serum FGF21 as an independent poor prognostic factor for overall survival (hazard ratio 2.20, *p* <0.001).

**Conclusions:**

Serum FGF21 may be a robust, non-invasive prognostic and treatment response marker for unresectable HCC treated with atezolizumab/bevacizumab.

**Impact and implications:**

FGF21 has been reported to act as a secreted immune-checkpoint factor, and elevated levels of FGF21 are associated with a poor prognosis in patients with HCC. It is not fully understood whether ICIs can overcome the impact of high FGF21 levels on the shortened prognosis of patients with HCC. In this multicentre retrospective study, patients with HCC and high baseline levels of serum FGF21 who received atezolizumab/bevacizumab treatment exhibited a significantly shorter overall survival and shorter progression-free survival. These findings suggest serum FGF21 as a robust prognostic marker and an indicator of treatment response in unresectable HCC treated with ICI-based therapy. These findings could be crucial for the implementation of personalised treatment strategies for unresectable HCC. However, identifying optimal therapeutic options for patients with unresectable HCC and high serum FGF21 levels remains an urgent and critical clinical issue.

## Highlights

• Tumour-secreted FGF21 functions as a secreted immune-checkpoint factor.
• High FGF21 is a robust prognostic marker in ICI-treated unresectable HCC.
• Elevated FGF21 predicts shorter OS and PFS in patients with ICI-treated HCC.
• FGF21 influences immune response and resistance to ICI therapy in HCC.
• Personalised therapy is crucial for patients with HCC with high serum FGF21.

## Graphical abstract

**Figure:**
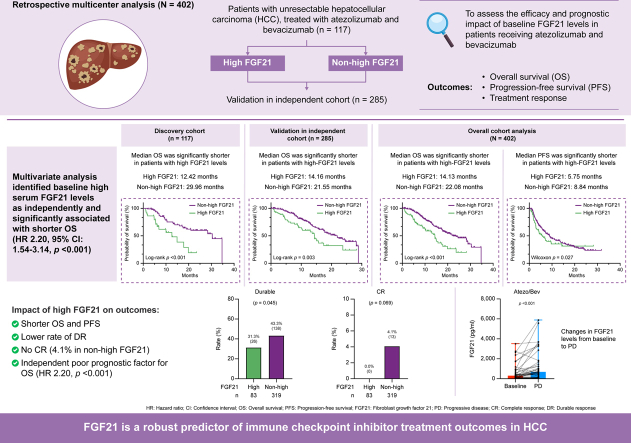

## Introduction

Primary liver cancer is the sixth most commonly diagnosed cancer worldwide and the third leading cause of cancer-related death, with ∼906,000 individuals diagnosed and 830,000 succumbing to the disease in 2020.^1^ Furthermore, the incidence of HCC is increasing.^2^ Therefore, identifying an optimal therapeutic option for patients with HCC, particularly unresectable HCC, is crucial. Recently, owing to successful clinical trials involving novel anti-cancer drugs for unresectable HCC, a range of systemic treatments have gained approval. These include multikinase inhibitors, such as sorafenib, lenvatinib, regorafenib, and cabozantinib, as well as monoclonal antibodies targeting vascular endothelial growth factor receptor 2 (VEGFR2), known as ramucirumab.[3], [4], [5] Finally, a combination therapy comprising the anti-VEGF-A antibody bevacizumab, programmed death ligand 1 (PD-L1) inhibitors atezolizumab and durvalumab, and the anti-cytotoxic T lymphocyte-associated antigen 4 (CTLA4) inhibitor tremelimumab has also been approved.^3^^,^^4^ Currently, these immune-checkpoint inhibitor (ICI)-based therapies are considered first-line treatments for unresectable HCC. Identifying prognostic and treatment response biomarkers for atezolizumab/bevacizumab is crucial in the clinical setting. Furthermore, with various therapeutic options available for unresectable HCC, identifying prognostic and treatment response biomarkers for all these therapies is clinically important to clarify the mechanisms underlying the poor prognosis of unresectable HCC.

Fibroblast growth factor 21 (FGF21) is a crucial metabolic regulator.^5^ It has a crucial role in maintaining energy balance and modulating carbohydrate and fat metabolism through both paracrine and endocrine mechanisms.^5^ The serum level of FGF21 is significantly elevated in several malignancies, including HCC.^6^ This high serum level could serve as a potential biomarker indicating poor prognosis in patients with HCC;^6^ however, the underlying mechanisms remain unclear. Nevertheless, a recent report suggested that FGF21 also functions as a secreted immune-checkpoint factor.^7^ Consequently, the suppressive effect of FGF21 on cancer immunity could lead to a poorer prognosis. In recent years, numerous therapeutic options for unresectable HCC, such as tyrosine kinase inhibitors (TKIs) and ICIs, have emerged. These therapies target specific molecules, including proteins involved in FGF receptor (FGFR)-mediated signalling and cancer immunity, and could affect the prognosis of patients with high serum FGF21 levels. However, whether high serum FGF21 levels affect patient prognosis or can serve as a prognostic biomarker for unresectable HCC treated with ICI-based therapy remains unclear.

Thus, in this study, we investigated the potential of FGF21 as a prognostic marker for unresectable HCC treated with ICI combination therapy involving atezolizumab/bevacizumab. We also explored whether traditional ICI-based therapy can effectively improve the prognosis of patients with high serum FGF21 levels, particularly those with HCC. Such insights enhance current understanding of the optimal therapeutic approach for this specific patient population.

## Patients and methods

### Patients and study design

In this retrospective study, we screened patients with unresectable HCC who were treated with atezolizumab/bevacizumab in the NORTE Study Group[8], [9], [10], [11], [12], [13] institutes between October 2020 and July 2024. Patients were included if they were treated with atezolizumab/bevacizumab, had properly preserved serum samples (collected immediately before treatment initiation) for analysing FGF21 levels, had adequate clinical data, and were evaluated for treatment response using enhanced computed tomography (CT) or magnetic resonance imaging (MRI) every 1–3 months. Patients were excluded if they did not have appropriately preserved serum samples for evaluating FGF21 levels or proper clinical data. To validate the results, we investigated data from independent studies involving 285 patients treated with atezolizumab/bevacizumab between October 2020 and July 2024 at six institutes other than NORTE Study Group institutes: Kagawa University Hospital, Japanese Red Cross Musashino Hospital, Yokohama City University Medical Center, Tokyo Medical and Dental University Hospital, University of Yamanashi Hospital, and NHO Nagasaki Medical Center. In addition, we evaluated the prognostic effects of baseline FGF21 levels in patients treated with sorafenib or lenvatinib in Hokkaido University Hospital between July 2009 and July 2024.

Baseline laboratory data included tumour markers, sex, age, HCC aetiology, Barcelona Clinic Liver Cancer (BCLC) stage, TNM classification, liver functional reserve assessment using the Child-Pugh classification, treatment protocol, treatment response, progression-free survival (PFS), and overall survival (OS). Treatment response was evaluated using the modified Response Evaluation Criteria in Solid Tumours (mRECIST) based on dynamic CT^14^ every 1–3 months. For the evaluation of treatment response, we examined the objective response rate (ORR), disease control rate (DCR), and rate of complete response (CR). In addition, we analysed the rate of durable response (patients with a durable partial response [PR] or stable disease [SD] for more than 6 months), which is a characteristic treatment response to ICI immunotherapy.^15^ Recent work indicates that patients with a durable response to atezolizumab/bevacizumab therapy for unresectable HCC demonstrated more favourable outcomes compared with those who did not respond to the therapy.^15^

The primary objective of this study was to analyse the prognostic effect and treatment response impact of baseline high FGF21 levels in patients with unresectable HCC treated with atezolizumab/bevacizumab. The secondary objective was to compare median OS, median PFS, and treatment response among patients with unresectable HCC with varying baseline serum FGF21 levels who were treated with the TKIs sorafenib and lenvatinib, or atezolizumab/bevacizumab (whole cohort).

This study adhered to the ethical principles outlined in the Declaration of Helsinki. The Hokkaido University Hospital Ethics Committee approved the study protocol (approval numbers: 017-0521, 020-0267, and 022-0052). Written informed consent was obtained from all enrolled patients who agreed to participate in the study. In addition, in cases where obtaining written informed consent for this study was not feasible, the Ethics Committee specifically approved the inclusion of patients who had provided broad consent for the use of their clinical samples and data, provided they did not explicitly decline participation in this study.

### Evaluation of serum FGF21 levels and their cut-off values

Serum FGF21 levels were analysed using ELISAs (R&D Systems, Minneapolis, MN, USA).^16^ Baseline serum FGF21 levels in patients treated with atezolizumab/bevacizumab were assessed using preserved serum samples and classified into two categories: high baseline FGF21 levels (high FGF21) and non-high baseline FGF21 levels (non-high FGF21) according to a previous report.^17^ To achieve this categorisation, patients were divided into the top 25% and others,^17^ with cut-off values established according to the discovery cohort of the NORTE study cohort. Subsequently, the cut-off value for the top 25% was used to divide the participants into a high FGF21 group and a non-high FGF21 group. We evaluated PFS and OS according to baseline FGF21 level. OS and PFS were compared between patients treated with atezolizumab/bevacizumab and those treated with lenvatinib or sorafenib.

### Atezolizumab/bevacizumab, sorafenib, and lenvatinib treatment protocols

In this study, patients with unresectable HCC were treated with 1,200 mg atezolizumab and 15 mg/kg bevacizumab every 3 weeks.^12^^,^^18^ Atezolizumab and/or bevacizumab were discontinued if the patients developed grade 3 or higher adverse events (AEs) or unacceptable AEs.

Sorafenib was administered orally at 400 mg twice daily, and lenvatinib was administered orally once daily, with the dose determined based on body weight as follows: 8 mg for patients <60 kg or 12 mg for patients ≥60 kg.^10^^,^[19], [20], [21] Treatments were discontinued if disease progression or unacceptable AEs were observed. The attending physician adjusted the doses of sorafenib and lenvatinib based on AEs and tolerability.

### Statistical analysis

Categorical variables were analysed using the chi-squared and Fisher’s exact tests. Continuous variables were assessed using Student *t* test or the Mann-Whitney *U* test.

Kaplan-Meier survival analysis was used to compare PFS and OS between groups. The survival curves were visually inspected to assess the plausibility of the proportional hazard assumption. Based on the observed patterns, both log-rank and generalised Wilcoxon tests were performed. The log-rank test was used to evaluate differences across the entire follow-up period, assuming proportional hazards. By contrast, the generalised Wilcoxon test was used to place greater emphasis on early events, because the survival curves showed noticeable divergence during the initial follow-up phase. Statistical significance was set at *p* <0.05 for all tests. Statistical analyses were performed using Prism 9.41 (GraphPad, La Jolla, CA, USA).

Multiple imputation (MI) was used to address missing data,. Specifically, 10 imputed datasets were generated under the assumption of missing at random. The MI procedure was performed using the ‘mice’ package in R (R Foundation for Statistical Computing, Vienna, Austria).

We enrolled 117 (NORTE cohort), 285 (validation cohort), or 402 (overall cohort) patients with or without high baseline serum FGF21 levels; to ensure a balanced assignment of patients, we performed propensity score matching (PSM). The matching process considered several variables, including baseline Barcelona Clinic Liver Cancer (BCLC) stage, Child-Pugh grade, sex, age, alanine aminotransferase (ALT), TNM stage, and the portal venous invasion (Vp) and alpha-fetoprotein (AFP) levels, which are considered to affect prognosis or significantly differ between the two groups. The adequacy of fit for the PSM approach was assessed using the Hosmer–Lemeshow test. Univariate Cox regression analysis was conducted for clinical factors and laboratory data, including tumour markers and serum FGF21 levels, and multivariate Cox regression analysis was conducted for factors showing significance (defined at *p* <0.05) in univariate analysis. Univariate and multiple Cox regression analyses and PSM were performed using EZR (Saitama Medical Centre, Jichi Medical University, Saitama, Japan).

## Results

### Patient characteristics and treatment responses

In the discovery cohort, we enrolled 117 consecutive patients with unresectable HCC who were treated with atezolizumab/bevacizumab between October 2020 and July 2024 at the institutes included in the NORTE Study Group and who met the inclusion criteria. Baseline patient characteristics are shown in Table 1. Of the patients, 78.6% (92/117) were men, with a median age of 71 years (range, 19–87 years), and 96.6% (113/117) of the patients had Child-Pugh grade A. A total of 0% (0/117), 41.0% (48/117), and 59.0% (69/117) of the patients had BCLC stages A, B, and C, respectively. In total, 29.1% (34/117) of patients showed an objective response.

**Table 1:** Comparison of baseline characteristics between patients with non-high and high FGF21 levels in the discovery and validation cohorts.

Characteristic	Discovery cohort^1^				Validation cohort			
	Total (n = 117)	Non-high FGF21 (n = 88)	High FGF21 (n = 29)	*p* value	Total (n = 285)	Non-high FGF21 (n = 231)	High FGF21 (n = 54)	*p* value
Age, years	71 (19–87)	72 (31–87)	70 (19–86)	0.72	74 (24–91)	73 (24–91)	78 (53–89)	0.02∗
Sex, n (%)								
Female	25 (21.4)	19 (21.6)	6 (20.7)	1	56 (19.6)	44 (19.0)	12 (22.2)	0.57
Male	92 (78.6)	69 (78.4)	23 (79.3)		229 (80.4)	187 (81.0)	42 (77.8)	
BMI, kg/m^2^	23.8 (9.8–35)	23.8 (15.5–35.0)	23.9 (9.8–33.0)	0.83	23.2 (14.4–41.6)	23.2 (15.0–41.6)	23.3 (14.4–36.6)	0.41
Aetiology, n (%)								
HBV	39 (33.3)	29 (33.0)	10 (34.5)	0.77	47 (16.5)	38 (16.5)	9 (16.7)	0.56
HCV	18 (15.4)	15 (17.0)	3 (10.3)		102 (35.8)	86 (37.2)	16 (29.6)	
NBNC	60 (51.3)	44 (50.0)	16 (55.2)		136 (47.7)	107 (46.3)	29 (53.7)	
Child-Pugh grade, n (%)								
A	113 (96.6)	84 (95.5)	29 (100.0)	0.571	240 (84.2)	199 (86.1)	41 (75.9)	0.11
B	4 (3.4)	4 (4.5)	0 (0.0)		44 (15.4)	31 (13.4)	13 (24.1)	
C					1 (0.4)	1 (0.4)	0 (0.0)	
TNM stage, n (%)								
2	14 (12.0)	14 (15.9)	0 (0.0)	0.06	34 (11.9)	25 (10.8)	9 (16.7)	0.358
3	34 (29.1)	22 (25.0)	12 (41.4)		122 (42.8)	104 (45.0)	18 (33.3)	
4A	22 (18.8)	17 (19.3)	5 (17.2)		39 (13.7)	31 (13.4)	8 (14.8)	
4B	47 (40.2)	35 (39.8)	12 (41.4)		90 (31.6)	71 (30.7)	19 (35.2)	
BCLC stage, n (%)								
A	0 (0.0)	0 (0.0)	0 (0.0)	1	8 (2.8)	8 (3.5)	0 (0.0)	0.12
B	48 (41.0)	36 (40.9)	12 (41.4)		131 (46.0)	111 (48.1)	20 (37.0)	
C	69 (59.0)	52 (59.1)	17 (58.6)		146 (51.2)	112 (48.5)	34 (63.0)	
ALBI grade, n (%)								
1	46 (39.3)	38 (43.2)	8 (27.6)	0.19	68 (23.9)	59 (25.5)	9 (16.7)	0.31
2	68 (58.1)	47 (53.4)	21 (72.4)		209 (73.3)	166 (71.9)	43 (79.6)	
3	3 (2.6)	3 (3.4)	0 (0.0)		8 (2.8)	6 (2.6)	2 (3.7)	
FIB-4 index	3.08 (0.87–24.04)	2.99 (0.87–24.04)	3.44 (1.00–15.17)	0.35	3.13 (0.09–32.80)	2.91 (0.09–32.80)	4.12 (0.12–15.75)	0.08
Best response, n (%)								
CR	1 (0.9)	1 (1.1)	0 (0.0)	0.336	12 (4.2)	12 (5.2)	0 (0.0)	0.012†
PR	33 (28.2)	26 (29.5)	7 (24.1)		74 (26.0)	58 (25.1)	16 (29.6)	
SD	54 (46.2)	38 (43.2)	16 (55.2)		100 (35.1)	89 (38.5)	11 (20.4)	
PD	28 (23.9)	23 (26.1)	5 (17.2)		56 (19.6)	39 (16.9)	17 (31.5)	
NA	1 (0.9)	0 (0.0)	1 (3.4)		43 (15.1)	33 (14.3)	10 (18.5)	
Biochemical analysis								
Plt, × 10^4^/μl	16.2 (3.6–46.4)	15.3 (3.6–46.4)	19.1 (6.1–41.9)	0.02∗	14.3 (3.7–44.9)	14.1 (3.7–44.9)	15.6 (8.4–40.3)	0.029∗
PT, %	93.1 (15.0-150)	93.9 (35.3–150.0)	92.7 (15.0–142.2)	0.93	91.9 (34.0–155.3)	91.2 (34.0–155.3)	92.0 (41.0–131.0)	0.94
Alb, g/dl	3.8 (2.7–4.8)	3.9 (2.7–4.8)	3.6 (3.0–4.5)	0.02∗	3.6 (2.1–4.7)	3.6 (2.1–4.7)	3.4 (2.4–5.4)	0.01∗
AST, IU/L	41 (11–672)	34 (11–162)	50 (14–672)	<0.01∗	41 (3–552)	39 (3–349)	54 (15–552)	<0.01∗
ALT, IU/L	27 (7–278)	26 (7–174)	33 (13–278)	0.08	28 (5–386)	28 (5–174)	27 (9-386)	0.49
T-Bil, mg/dl	0.8 (0.3–2.9)	0.8 (0.3–2.9)	0.8 (0.5–2.6)	0.93	0.8 (0.3–3)	0.8 (0.3–2.4)	0.8 (0.4–3)	0.20
AFP, ng/ml	83.4 (0.8–290,835)	77.2 (0.8–200,000.0)	138.8 (2.9–290,835)	0.08	82.8 (0.5–1,133,450.0)	89.2 (1–1,133,450)	77.7 (0.5–628,992)	0.326

Data are expressed as median (range) unless otherwise indicated and analysed by Mann-Whitney U test unless indicated.

AFP, alpha-fetoprotein; Alb, albumin; ALBI, albumin–bilirubin; ALT, alanine aminotransferase; AST, aspartate aminotransferase; BCLC, Barcelona Clinic Liver Cancer; CR, complete response; FIB-4, fibrosis-4 index; HR, hazard ratio; NA, not available; NBNC, non-HBV non-HCV; PD, progressive disease; Plt, platelet count; PR, partial response; PT, prothrombin time; SD, stable disease; T-Bil, total bilirubin.

∗ Mann-Whitney *U* test.

† Fisher’s exact test.

### Association among baseline serum FGF21 levels, PFS, and OS in patients with unresectable HCC treated with atezolizumab/bevacizumab in the discovery cohort

Subsequently, we analysed the baseline FGF21 levels of these 117 patients and classified them into high FGF21 (top 25%) and non-high FGF21 (75%) groups.^17^ The cut-off values were set at 914.8 pg/ml, and patients with baseline FGF21 levels ≥915 pg/ml were defined as patients with high FGF21.

First, we compared the baseline patient characteristics between patients with and without high FGF21. Although baseline age, Child-Pugh grade, aetiology of HCC, and BCLC stage were similar between these groups, aspartate aminotransferase levels (*p* <0.01) and platelet counts (*p* = 0.02) were significantly higher in patients with high FGF21 than in those without. Baseline albumin levels (*p* = 0.01) were significantly lower in patients with high FGF21 than in those without (Table 1).

**Fig. 1:**
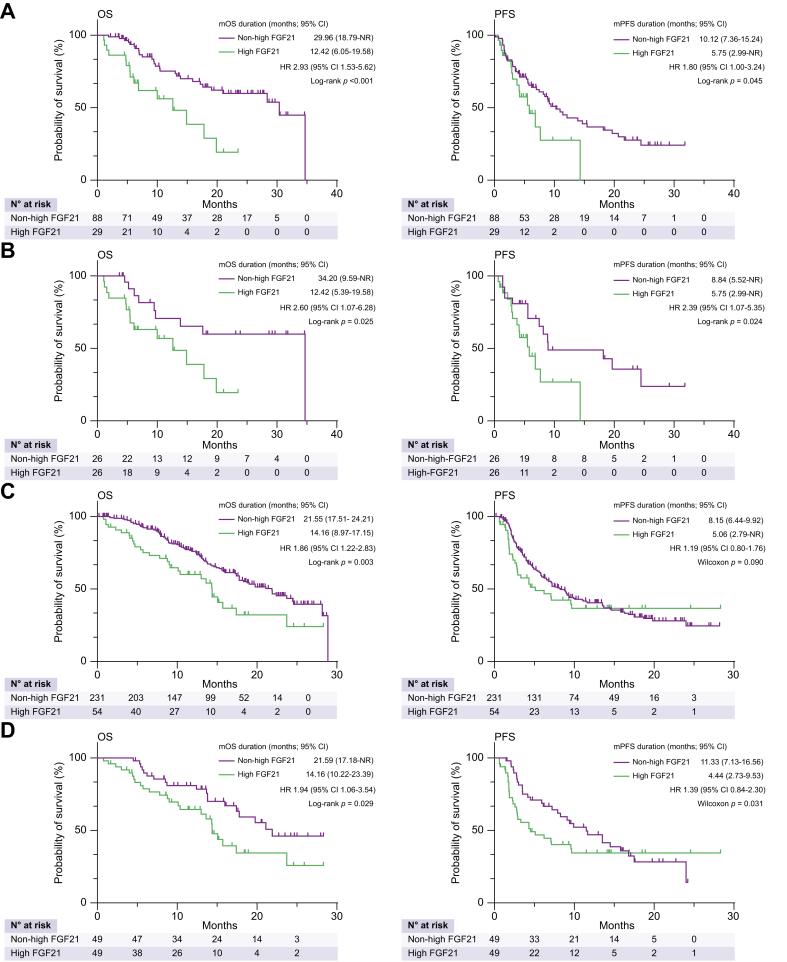
OS and PFS of patients treated with atezolizumab and bevacizumab and stratified according to baseline serum FGF21 levels. (A,B) Discovery cohort. (A) OS: level of significance, *p* <0.001 (log-rank test); PFS, level of significance, *p* = 0.045 (log-rank test). (B) Patients were categorised using propensity scores (BCLC stage, Child-Pugh grade, sex, age, ALT, TNM stage, presence of Vp, and AFP levels). OS, level of significance: *p* = 0.025 (log-rank test); PFS, level of significance: *p* = 0.024 (log-rank test). (C,D) Validation cohort. (C) OS, level of significance: *p* = 0.003 (log-rank test); PFS, level of significance: *p* = 0.090 (generalised Wilcoxon test). (D) Patients were categorised using propensity scores (BCLC stage, Child-Pugh grade, sex, age, ALT, TNM stage, presence of Vp, and AFP levels). OS, level of significance: *p* = 0.029 (log-rank test); PFS, level of significance: *p* = 0.031 (generalised Wilcoxon test). AFP, alpha-fetoprotein; ALT, alanine aminotransferase; BCLC, Barcelona Clinic Liver Cancer; high FGF21, high baseline FGF21 levels; HR, hazard ratio; non-high FGF21, non-high baseline FGF21 levels; NR, not reached; (m)OS, (median) overall survival; (m)PFS, (median) progression-free survival.

Next, we compared the median OS and PFS of patients with and without high FGF21. Median OS was significantly shorter in patients with high FGF21 than in those with non-high FGF21 (median OS: 12.42 [95% CI 6.05–19.58] *vs.* 29.96 [95% CI, 18.79–not reached [NR]) months; *p* <0.001; Fig. 1A]. PFS was significantly shorter in patients with high FGF21 than in those with non-high FGF21 (PFS: 5.75 [95% CI, 2.99–NR] *vs.* 10.12 [95% CI, 7.36–15.24] months; *p* = 0.045; Fig. 1A).

Subsequently, to ensure a balanced assignment of patients with or without high FGF21, 1:1 PSM was performed. The matching process considered baseline BCLC stage, Child-Pugh grade, sex, age, ALT, TNM stage, presence of Vp, and AFP levels, which are factors known to affect OS and may significantly differ between patients with or without high FGF21. A comparison of baseline patient characteristics between 52 selected propensity score-matched patients with or without high FGF21 showed that these characteristics were well matched between the groups (Hosmer–Lemeshow test, *p* = 0.74; Table S1). Median OS was significantly shorter in patients with high FGF21 than in those with non-high FGF21 (median OS: 34.2 months [95% CI, 9.59–NR] *vs.* 12.42 [95% CI, 5.39–19.58] months; *p* = 0.025; Fig 1B). Similarly, median PFS was significantly shorter in patients with high FGF21 than in those with non-high FGF21 (median PFS: 8.84 months [95% CI, 5.52–NR] *vs.* 5.75 [95% CI, 2.99–NR] months; *p* = 0.024; Fig. 1B).

### Independent validation in 285 patients with HCC who were treated with atezolizumab/bevacizumab

Subsequently, these results were validated in an independent cohort comprising 285 patients with HCC who were treated with atezolizumab/bevacizumab. Baseline patient characteristics are shown in Table 1. Median OS was also significantly shorter in patients with high FGF21 than in those without (median OS, 14.16 [95% CI, 8.97–17.15] *vs.* 21.55 [95% CI, 17.51–24.21] months; *p* = 0.003; Fig. 1C). Median PFS was marginally significantly shorter in patients with high FGF21 than in those with non-high FGF21 (median PFS, 8.15 [95% CI, 6.44–9.92] *vs.* 5.06 [95% CI, 2.79–NR] months, respectively; *p* = 0.09; Fig. 1C). A comparison of baseline patient characteristics between selected propensity score-matched patients (49 with and 49 without high FGF21) showed that these characteristics were well matched in the two groups (Hosmer–Lemeshow test, *p* = 0.72; Table S2). Median OS was also significantly shorter in patients with high FGF21 than in those without (median OS, 14.16 [95% CI, 10.22–23.39] *vs.* 21.59 [95% CI, 17.18–NR] months, respectively; *p* = 0.029; Fig. 1D). In addition, median PFS was significantly shorter in patients with high FGF21 than in those with non-high FGF21 (median PFS, 4.44 [95% CI, 2.73–9.53] *vs.* 11.33 months [95% CI, 7.13–16.56] months, respectively; *p* = 0.031; Fig. 1D).

### Serum FGF21 levels as a prognostic factor and predictor of treatment response for unresectable HCC in the overall cohort following treatment with atezolizumab/bevacizumab

**Fig. 2:**
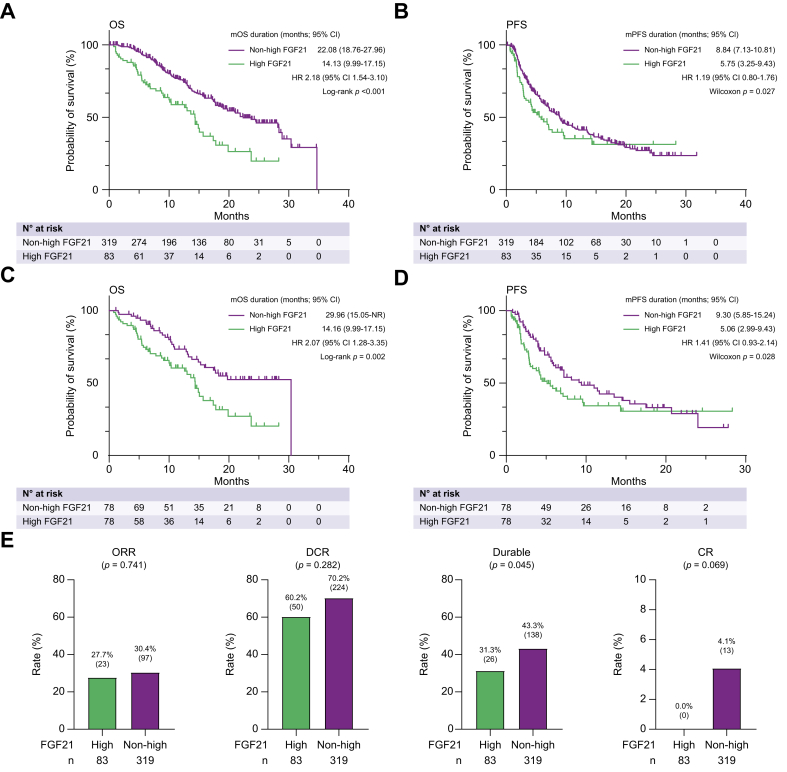
OS, PFS, and treatment response of patients treated with atezolizumab and bevacizumab and stratified according to baseline serum FGF21 levels in the whole cohort. (A) OS, level of significance: *p* <0.001 (log-rank test). (B) PFS, level of significance: *p* = 0.027 (generalised Wilcoxon test). (C,D) Patients with unresectable HCC treated with atezolizumab and bevacizumab were categorised using propensity scores, including BCLC stage, Child-Pugh grade, sex, age, ALT, TNM stage, presence of Vp, and AFP levels. (C) OS, level of significance: *p* = 0.002 (log-rank test). (D) PFS, level of significance: *p* = 0.028 (generalised Wilcoxon test). (E) ORR, DCR, durable rate, and CR rat, level of significance: *p* = 0.741, *p* = 0.282, *p* = 0.045, and *p* = 0.069, respectively (Fisher’s exact test). Durable is defined as durable response of PR or SD of >6 months, or CR. CR, complete response; DCR, disease control rate; high FGF21, high baseline FGF21 levels; HR, hazard ratio; non-high FGF21, non-high baseline FGF21 levels; ORR, objective response rate; (m)OS, (median) overall survival; (m)PFS, (median) progression-free survival; PR, partial response; SD, stable disease.

In the overall cohort (N = 402; Table 2), median OS was significantly shorter in patients with high FGF21 than in those without (median OS, 14.13 [95% CI, 9.99–17.15] *vs.* 22.08 [95% CI, 18.76–27.96] months, respectively; *p* <0.001; Fig. 2A). In addition, median PFS was significantly shorter in patients with high FGF21 than in those without high FGF21 (median PFS, 5.75 [95% CI, 3.25–9.43] *vs.* 8.84 months [95% CI, 7.13–10.81] months, respectively; *p* = 0.027; Fig. 2B).

**Table 2:** Comparison of baseline characteristics between patients with non-high and high FGF21 levels in the overall cohort.

Characteristic	Total (N = 402)	Non-high FGF21 (n = 319)	High FGF21 (n = 83)	*p* value
Age, years	73 (19–91)	73 (24–91)	74 (19–89)	0.1
Sex, n (%)				
Female	81 (20.1)	63 (19.7)	18 (21.7)	0.76
Male	321 (79.9)	256 (80.3)	65 (78.3)	
BMI, kg/m^2^	23.4 (9.8–41.6)	23.4 (15.0–41.6)	23.4 (9.8–36.6)	0.53
Aetiology, n (%)				
HBV	86 (21.4)	67 (21.0)	19 (22.9)	0.30
HCV	120 (29.9)	101 (31.7)	19 (22.9)	
NBNC	196 (48.8)	151 (47.3)	45 (54.2)	
Child-Pugh grade, n (%)				
A	353 (87.8)	283 (88.7)	70 (84.3)	0.41
B	48 (11.9)	35 (11.0)	13 (15.7)	
C	1 (0.2)	1 (0.3)	0 (0.0)	
BCLC stage, n (%)				
A	8 (2.0)	8 (2.5)	0 (0.0)	0.15
B	179 (44.5)	147 (46.1)	32 (38.6)	
C	215 (53.5)	164 (51.4)	51 (61.4)	
TNM				
2	48 (11.9)	39 (12.2)	9 (10.8)	0.89
3	156 (38.8)	126 (39.5)	30 (36.1)	
4A	61 (15.2)	48 (15.0)	13 (15.7)	
4B	137 (34.1)	106 (33.2)	31 (37.3)	
Vp				
(–)	342 (85.1)	278 (87.1)	64 (77.1)	0.04
(+)	60 (14.9)	41 (12.9)	19 (22.9)	
ALBI grade, n (%)				
1	114 (28.4)	97 (30.4)	17 (20.5)	0.19
2	277 (68.9)	213 (66.8)	64 (77.1)	
3	11 (2.7)	9 (2.8)	2 (2.4)	
FIB-4 index	3.08 (0.09–32.80)	2.93 (0.09–32.80)	3.83 (0.12–15.75)	0.04
Best response, n (%)				
CR	13 (3.2)	13 (4.1)	0 (0.0)	0.15
PR	107 (26.6)	84 (26.3)	23 (27.7)	
SD	154 (38.3)	127 (44.4)	27 (37.5)	
PD	84 (20.9)	127 (39.8)	27 (32.5)	
NA	44 (10.9)	33 (10.3)	11 (13.3)	
Biochemical analysis				
Plt, × 10^4^/μl	16.6 (3.6–426.0)	14.3 (3.6–46.4)	16.8 (6.1–41.9)	<0.01
PT, %	92.0 (15.0–155.3)	92.0 (34–155.3)	92.0 (15–142.2)	0.99
Alb, g/dl	3.6 (2.1–4.8)	3.7 (2.1–4.8)	3.5 (2.4–4.5)	<0.01
AST, IU/L	41 (3–672)	38 (3–349)	54 (14–672)	<0.01
ALT, IU/L	28 (5–386)	28 (5–174)	30 (9–386)	0.1
T-Bil, mg/dl	0.8 (0.3–3)	0.8 (0.3–2.9)	0.8 (0.4–3)	0.22
AFP, ng/ml	82.8 (0.5–1,133,450)	80.1 (0.8–113,345)	82.8 (0.5–628,992.0)	0.07

Data are expressed as median (range) unless otherwise indicated.

ALBI, albumin–bilirubin; ALT, alanine aminotransferase; AST, aspartate aminotransferase; BCLC, Barcelona Clinic Liver Cancer; BMI, body mass index; CR, complete response; FGF21, fibroblast growth factor 21; HBV, hepatitis B virus; HCV, hepatitis C virus; NBNC, non-HBV non-HCV; PD, progressive disease; PR, partial response; SD, stable disease.

FIB-4 index; level of significance: *p* = 0.04 (Mann-Whitney U-test), Platelet counts; level of significance: *p* < 0.01 (Mann-Whitney U-test), AST levels; level of significance: *p* < 0.01 (Mann-Whitney U-test), Albumin levels; level of significance: *p* < 0.01 (Mann-Whitney U-test).

**Fig. 3:**
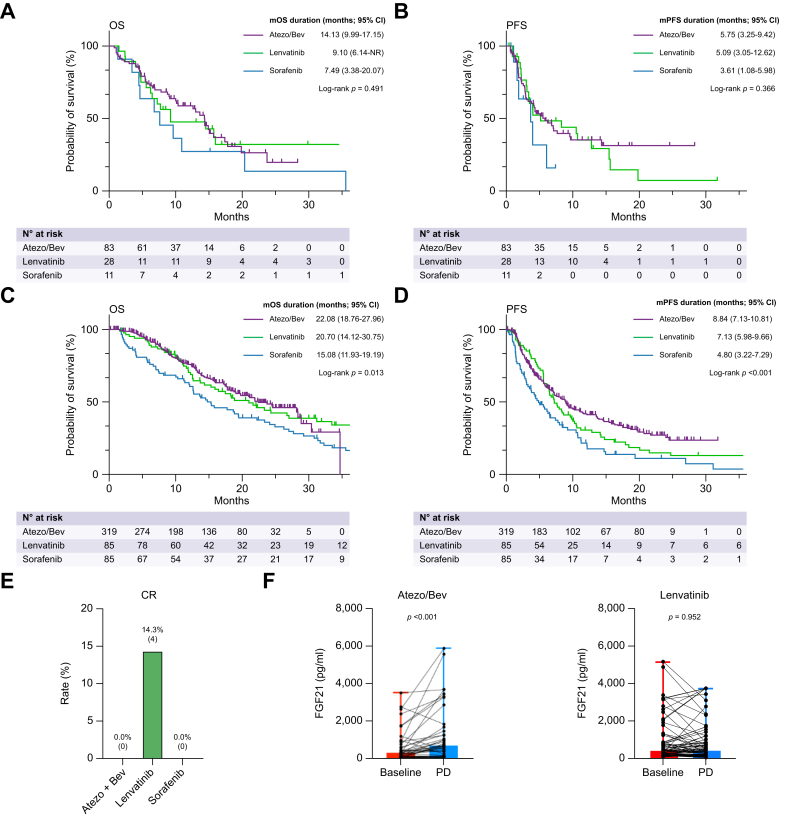
Comparison of OS, PFS, and treatment response among patients treated with atezolizumab/bevacizumab, lenvatinib, or sorafenib, stratified according to baseline serum FGF21 levels. (A,B) Kaplan–Meier estimates of OS and PFS stratified by treatment regimen in patients with high baseline FGF21 levels (high FGF21). (A) OS, level of significance: *p* = 0.491 (log-rank test). (B) PFS, level of significance: *p* = 0.366 (log-rank test). (C,D) Kaplan–Meier estimates of OS and PFS stratified by treatment regimen in patients with non-high baseline FGF21 levels (non-high FGF21). (C) OS, level of significance: *p* = 0.013 (log-rank test). (D) PFS, level of significance: *p* <0.001 (log-rank test). (E) Comparison of CR rate among atezolizumab/bevacizumab, lenvatinib, or sorafenib in patients with high FGF21. Lenvatinib was the only treatment to achieve CR in four cases. (F) Changes in FGF21 levels from baseline to PD. Atezolizumab/bevacizumab, *p* <0.001; lenvatinib, *p* = 0.952. (Wilcoxon matched-pairs signed rank test). Atezo/Bev, atezolizumab/bevacizumab; CR, complete response; high FGF21, high baseline FGF21 levels; HR, hazard ratio; non-high FGF21, non-high baseline FGF21 levels; (m)OS, (median) overall survival; PD, progressive disease; (m)PFS, (median) progression-free survival.

Next, we performed 1:1 PSM, considering baseline BCLC stage, Child-Pugh grade, sex, age, ALT, TNM stage, presence of Vp, and AFP levels (Hosmer–Lemeshow test, *p* = 0.14; Table S3). Median OS was also significantly shorter in patients with high FGF21 than in those without (median OS, 14.16 [95% CI, 9.99–17.15] *vs.* 29.96 [95% CI, 15.05–NR] months, respectively; *p* = 0.002; Fig. 2C). In addition, PFS was also significantly shorter in patients with high FGF21 than in those with non-high FGF21 (PFS, 5.06 [95% CI, 2.99–9.43] *vs.* 9.30 [95% CI, 5.85–15.24] months; *p* = 0.028; Fig. 3D).

Multivariate Cox regression analysis of factors associated with prognosis in patients who were treated with atezolizumab/bevacizumab revealed a high FGF21 (hazard ratio [HR)], 2.20; 95% CI, 1.54–3.14; *p* <0.001), Child-Pugh grade B and C (HR, 3.28; 95% CI, 2.19–4.90; *p* <0.001), AFP >82.8 (HR, 1.81; 95% CI, 1.30–2.53; *p* <0.001), and FIB-4 index >3.08 (HR, 1.40; 95% CI, 1.01–1.94; *p* = 0.044) to be independently and significantly associated with shorter OS (Table 3).

**Table 3:** Univariate and multivariate Cox regression analyses of factors associated with prognosis.

Characteristic	Univariate analysis		Multivariate analysis	
	HR (95% CI)	*p* value	HR (95% CI)	*p* value
Age >73, years	1.12 (0.821–1.52)	0.480		
Male	0.96 (0.67–1.39)	0.831		
Child-Pugh grade B, C	4.04 (2.75–5.94)	<0.001	3.28 (2.19–4.90)	<0.001
ALBI grade 2, 3	2.20 (1.48–3.29)	<0.001		
BCLC stage C	1.61 (1.17–2.20)	0.003	1.23 (0.88–1.72)	0.219
FIB-4 index >3.08	1.67 (1.22–2.29)	0.001	1.40 (1.01–1.94)	0.044
AFP >82.8, ng/ml	2.13 (1.55–2.92)	<0.001	1.81 (1.30–2.53)	<0.001
FGF21 >915, pg/ml	2.18 (1.54–3.10)	<0.001	2.20 (1.54–3.14)	<0.001
BMI <23.4, kg/m^2^	1.52 (1.12–2.08)	0.008	1.457 (1.06–2.00)	0.019
Platelet, × 10^4^/μl	1.11 (0.82–1.51)	0.480		

AFP, alpha-fetoprotein; ALBI, albumin–bilirubin; BCLC, Barcelona Clinic Liver Cancer; FIB-4, fibrosis-4 index; HR, hazard ratio.

### Association among baseline serum FGF21 levels and treatment response in patients with unresectable HCC treated with atezolizumab/bevacizumab

Subsequently, we compared treatment responses among patients with and without high FGF21, evaluating ORR, DCR, CR rate, and durable response (patients achieving durable SD, durable PR [response sustained for more than 6 months]),^15^ or CR rate. Although patients with high baseline serum FGF21 levels had lower ORR and DCR, these differences were not statistically significant (Fig. 2E). However, the durable response rate was significantly lower in this group than in the non-high FGF21 group (*p* = 0.045). Moreover, among patients with high FGF21, none achieved a CR, whereas 4.1% (13/319) of patients with baseline non-high serum FGF21 levels achieved CR.

### Association among baseline serum FGF21 levels and treatment response and prognosis in patients with unresectable HCC treated with sorafenib or lenvatinib

Next, we analysed the association between baseline serum FGF21 levels and PFS and OS rates in patients treated with lenvatinib or sorafenib. The baseline characteristics of the patients treated with sorafenib are shown in Table S4.

Median OS rates tended to be shorter in patients with high FGF21 than in those without (median OS, 7.49 [95% CI, 3.38–20.07] *vs.* 15.08 [95% CI, 11.93–19.19] months, respectively; *p* = 0.075; Fig. S1A). Median PFS was similar between patients with and without high FGF21 (Fig. S1B).

The baseline characteristics of the patients treated with lenvatinib are shown in Table S5. Median OS rates were significantly shorter in patients with high FGF21 than in those without (median OS, 9.10 [95% CI, 6.14–NR] *vs.* 20.70 [95% CI, 14.12–30.75] months, respectively; *p* = 0.049; Fig. S1C). By contrast, median PFS was similar between patients with and without high FGF21 (Fig. S1D).

### Comparison of prognosis and treatment response among patients with or without high FGF21, treated with lenvatinib, sorafenib, or atezolizumab/bevacizumab

Next, we compared the median OS rates among patients with high FGF21 who were treated with sorafenib, lenvatinib, and atezolizumab/bevacizumab in the overall cohort and the first-line limited cohort (Tables S6 and S7). In the overall cohort, median OS rates were similar among patients with high FGF21 treated with atezolizumab/bevacizumab (n = 83), lenvatinib (n = 28), or sorafenib (n = 11) (median OS, 14.13 [95% CI, 9.99–17.15], 9.10 [95% CI, 6.14–NR], and 7.49 [95% CI, 3.38–20.07] months for atezolizumab/bevacizumab, lenvatinib, and sorafenib, respectively; *p* = 0.491; Fig. 3A). Median PFS was similar among the three groups (Fig. 3B). Similar results were observed in the first-line limited cohort (Fig. S2A,B).

Subsequently, we compared the median OS rates among patients with non-high FGF21 who were treated with sorafenib, lenvatinib, or atezolizumab/bevacizumab in the overall cohort and first-line limited cohort (Tables S8 and S9). In the overall cohort, median OS rates were significantly different among patients with non-high FGF21 treated with atezolizumab/bevacizumab (n = 319), lenvatinib (n = 85), or sorafenib (n = 85) (median OS, 22.08 [95% CI, 18.76–27.96], 20.70 [95% CI, 14.12–30.75], and 15.08 [95% CI, 11.93–19.19] months for atezolizumab/bevacizumab, lenvatinib, and sorafenib, respectively; *p* = 0.013; Fig. 3C). Median PFS rates were similarly different among these three groups (median PFS, 8.84 [95% CI, 7.13–10.81], 7.13 [95% CI, 5.98–9.66], and 4.80 [95% CI, 3.22–7.29] months for atezolizumab/bevacizumab, lenvatinib, and sorafenib, respectively; *p* <0.001; Fig. 3D). Similar results were observed in the first-line limited cohort (Fig. S2C, D).

In addition, among patients with high baseline FGF21 levels, patients treated with lenvatinib alone could achieve CR (14.8%, 4/28), whereas none of the patients treated with atezolizumab/bevacizumab (0%, 0/83) and sorafenib (0%, 0/11) achieved CR (Fig. 3E).

### Changes in FGF21 levels between baseline and the point of disease progression in HCC patients treated with atezolizumab/bevacizumab or lenvatinib

Finally, we compared the changes in serum FGF21 levels from baseline to the point of progressive disease (PD) in patients treated with atezolizumab/bevacizumab or lenvatinib. This analysis included patients with paired preserved serum samples available at both baseline and PD time points. Among patients treated with atezolizumab/bevacizumab (n = 41), serum FGF21 levels were significantly increased at the point of PD compared with baseline levels (*p* <0.001) (Fig. 3F). By contrast, patients treated with lenvatinib (n = 73) experienced no significant change in serum FGF21 levels from baseline to the PD time point.

## Discussion

Recently, it was reported that secreted FGF21 may influence cancer immunity. However, to the best of our knowledge, it remains unclear whether FGF21 affects treatment response and prognosis, or whether it can serve as a prognostic biomarker for unresectable HCC treated with ICI-based therapy. In this study, high FGF21 was a strong poor prognostic factor in patients with unresectable HCC treated with atezolizumab/bevacizumab-based therapy. Moreover, patients with high FGF21 exhibited significantly shorter PFS and a significantly lower rate of durable response (including durable SD, PR, and CR) compared to those with non-high FGF21. Furthermore, none of the patients with high FGF21 achieved CR.

In this multicentre study, median OS rates were significantly lower in patients with high FGF21 than in those from the discovery cohort. Even after PSM by BCLC stage, Child-Pugh grade, sex, age, ALT, TNM stage, presence of Vp, and AFP levels, median OS was significantly shorter in patients with high FGF21 than in those without. Analysis of an independent cohort of 285 patients validated these results. Finally, in the overall cohort of 402 cases, median OS rates were significantly shorter in patients with high FGF21 than in those without. Multivariate Cox regression analysis of factors associated with OS in patients treated with atezolizumab/bevacizumab revealed that, in addition to the FIB-4 index, AFP, BMI, and Child-Pugh grade, high FGF21 was independently and significantly associated with lower OS rates. To the best of our knowledge, this is the first report suggesting FGF21 as a marker for poor prognosis in the ICI-based treatment of malignancies, such as HCC.

In the IMbrave150 clinical trials, a combination of atezolizumab/bevacizumab considerably prolonged median OS compared with that observed with the previous standard sorafenib treatment.^22^ Thus, atezolizumab/bevacizumab are first-line therapies for patients with unresectable HCC. Recently, it was reported that, in a pathological analysis, baseline immunity status, including a high T effector signature, high intratumoural CD8^+^ T cell infiltration, and a reduced regulatory:effector T cell ratio, was significantly associated with higher OS rates.^23^ Thus, the immune status of HCC and related biomarkers^10^ is associated with prognosis in patients with unresectable HCC treated with atezolizumab/bevacizumab. To our knowledge, the present study is the first to report the association between FGF21, which is well known as a metabolic regulator,^5^ and the prognosis of combined treatment with atezolizumab/bevacizumab.

Importantly, high FGF21 is associated with poor prognosis in various malignancies, including HCC.^6^ In our study, the median PFS in patients with high FGF21 was significantly shorter compared with that in patients without. Thus, high FGF21 is associated with both poor prognosis and poor treatment response in patients with HCC treated with atezolizumab/bevacizumab. Moreover, recent reports indicate that achieving a durable response (defined as sustained therapeutic efficacy lasting >6 months) is crucial for prolonging survival of patients with unresectable HCC receiving atezolizumab/bevacizumab treatment.^15^ Durable response is a treatment characteristic unique to ICIs.^15^ In the present study, although the ORR and DCR were slightly lower in patients with high FGF21, the difference was not statistically significant. However, durable response rates (durable PR and SD, and CR) were significantly lower in patients with high FGF21, suggesting that ICI efficacy is suboptimal in this subgroup. Given that atezolizumab/bevacizumab is a combination therapy of an anti-VEGF antibody and ICI, the ORR could have been influenced by the effects of bevacizumab. By contrast, the ICI-specific durable response might have been impacted by tumour immunosuppressive effects mediated by elevated FGF21 levels. This potential mechanism could also explain the absence of any patients achieving CR, a therapeutic outcome requiring profound antitumor activity. These findings suggest that the efficacy of ICIs alone is not adequate in patients with high FGF21 levels. By contrast, lenvatinib, a TKI capable of blocking the FGF21/FGFR1 axis,^24^ demonstrated comparable PFS between patients with high and non-high FGF21 levels. Furthermore, among patients treated with sorafenib, lenvatinib, or atezolizumab/bevacizumab, the cases of CR observed in the high FGF21 subgroup were exclusively associated with lenvatinib. In addition, during the development of resistance to ICI-based therapy, FGF21 levels were significantly elevated (Fig. 3F). This supports the hypothesis that elevated FGF21 levels may contribute to resistance to ICI-based therapy. By contrast, during lenvatinib treatment, FGF21 levels remained similar between the baseline and the PD point. This suggests treatments targeting the FGF21/FGFR1 axis, such as lenvatinib, in combination with ICIs, as effective therapeutic strategies for patients with high FGF21 levels.

Until recently, the role of FGF21 in cancer immunity remained unexplored. However, recent findings suggested that FGF21 has a crucial role in suppressing cancer immune responses.^7^ When secreted by tumours, FGF21 significantly disrupts the anti-cancer ability of the immune system by altering cholesterol metabolism within CD8^+^ T cells. This involves the ability of FGF21 to sustain increased activity in the AKT-mTORC1-SREBP1 signalling pathway in T cells, which in turn increases cholesterol biosynthesis and leads to T cell exhaustion. Counteracting FGF21 overexpression through knockdown techniques or the use of neutralising antibodies can restore the balance in the AKT-mTORC1 signalling pathway and reduce cholesterol accumulation in CD8^+^ T cells, resulting in the enhanced anti-cancer ability of T cells and markedly reduced tumour growth.^7^ Thus, FGF21 is considered a secreted immune-checkpoint protein that alters cholesterol metabolism in CD8^+^ T cells. The results of this study also indicated that cancer cells secreting high levels of FGF21 were resistant to anti-PD-1 monotherapy, whereas the combination of anti-FGF21 and anti-PD-1 therapies showed significant anti-cancer effects.^7^ This result might be consistent with our finding that a high FGF21 level is associated with poor prognosis in patients with unresectable HCC who were treated with an anti-PDL-1 combination therapy of atezolizumab/bevacizumab, which does not affect FGF21-mediated signalling.

Alternative hypotheses may also be considered regarding the observation that increased serum FGF21 levels are associated with poor prognosis in patients with HCC. In several malignancies, including colorectal cancer,^25^ urothelial carcinoma,^26^ thyroid cancer,^27^ endometrial cancer,^28^ and HCC,^6^ serum FGF21 levels are significantly elevated and associated with recurrence or advanced stages. In addition, FGF21 contributes to the increased malignant potential of papillary thyroid carcinoma cells by activating the FGFR signalling pathway and enhancing epithelial-to-mesenchymal transition (EMT) signals.^27^ *In vitro* studies on HCC cell lines showed that FGF21 inhibits metastasis by repressing EMT driven by β-catenin signalling. However, when β-Klotho (KLB) was suppressed and FGF21 was simultaneously overexpressed, HCC cell mobility and activation of genes associated with EMT induction increased.^29^ Thus, in the absence of KLB, overexpression of FGF21 might cause EMT. Moreover, there is a possible link between FGF21 expression and the development of sorafenib resistance in HCC. FGF21 interacts with NF-E2-related factor 2 (NRF2) at its C-terminal region. This interaction reduces the ubiquitination of NRF2, thereby stabilising it. This creates a positive feedback mechanism in sorafenib-resistant HCC cells via an enhanced antioxidant stress response.^30^

FGF21 knockdown in a mouse model was shown to accelerate HCC development.^31^ In addition, prolonged administration of FGF21 inhibits the onset of HCC induced by chemical agents.^32^ Moreover, FGF21 is a potential therapeutic target in non-alcoholic fatty liver disease, and clinical trials on FGF21 in this condition have reported favourable results.^33^ Therefore, further investigations are required to elucidate the distinct roles of FGF21 in HCC initiation and proliferation.

Recently, in sorafenib or lenvatinib treatment of patients with unresectable HCC, patients with high FGF21 reportedly showed significantly lower OS rates compared with those without such levels.^17^ Thus, it is crucial to determine whether a novel therapeutic option involving atezolizumab/bevacizumab can address this issue. In this study, the median OS of patients with HCC and high FGF21 treated with atezolizumab/bevacizumab was significantly shorter compared with that of patients without, even after PSM and in multivariate Cox regression analyses. Moreover, the median OS was similar among patients with high FGF21 who were treated with sorafenib, lenvatinib, or atezolizumab/bevacizumab. Therefore, there is an urgent need to identify effective therapeutic options for patients with elevated serum FGF21 levels. It is essential to determine whether other combinations of ICIs can overcome this challenge. Furthermore, a combination of anti-FGF21 therapy, including lenvatinib, and ICIs may be necessary for patients with unresectable HCC who have high FGF21 levels.

Recently, studies reported increased FGF21 levels in patients with advanced liver disease, including decompensated liver cancer.^34^^,^^35^ Nevertheless, in the present study, the prevalence of high FGF21 levels was similar across patients regardless of Child-Pugh grade (Fig. S3A). Given that this study focussed on unresectable HCC, we speculate that the increased FGF21 levels are primarily attributed to tumour secretion, with only limited association with a compromised hepatic reserve. In addition, the subgroup analyses of patients with Child-Pugh grade A in the discovery, validation, and whole cohorts had similar results to those of the overall analysis of patients in these cohorts (Fig. S3B–G).

This study is limited by its retrospective design and the limited number of patients treated with sorafenib and lenvatinib. Therefore, a large-scale prospective study is needed to validate these findings. Furthermore, the determination of an appropriate cut-off value should be based on a larger cohort of patients with unresectable HCC.

## Conclusions

This study is the first to reveal that high FGF21 could serve as a robust and non-invasive prognostic and treatment response marker for unresectable HCC treated with the ICI-based therapy of atezolizumab/bevacizumab. These results could be vital for the implementation of personalised treatment strategies for unresectable HCC. However, determining the optimal therapeutic option for patients with unresectable HCC and high FGF21 is an urgent and critical clinical issue.

## Abbreviations

AE, adverse event; AFP, alpha-fetoprotein; Alb, albumin; ALBI, albumin–bilirubin; ALT, alanine aminotransferase; AST, aspartate aminotransferase; BCLC, Barcelona Clinic Liver Cancer; CR, complete response; CTLA4, cytotoxic T lymphocyte-associated antigen 4; DCR, disease control rate; EMT, epithelial-to-mesenchymal transition; FGF21, fibroblast growth factor 21; FGFR, FGF receptor; FIB-4, Fibrosis-4; HBFL, high baseline FGF21 level; HCC, hepatocellular carcinoma; HR, hazard ratio; ICI, immune-checkpoint inhibitor; KLB, β-Klotho; MI, multiple imputation; mRECIST, modified Response Evaluation Criteria in Solid Tumours; NBNC, non-HBV non-HCV; NR, not reached; ORR, objective response rate; OS, overall survival; PD, progressive disease; PD-L1, programmed death ligand 1; PD, XXXX;PFS, progression-free survival; Plt, platelet count; PSM, propensity score matching; PT, prothrombin time; T-Bil, total bilirubin; TKI, tyrosine kinase inhibitor; VEGFR2, vascular endothelial growth factor receptor 2; Vp, venous phase.

## Financial support

This study was supported, in part, by grants from the 10.13039/100009619Japan Agency for Medical Research and Development (AMED; grants no. JP24fk0210126, JP24fk0310501, JP24fk0210121, JP24fk0210112, JP24fk0210142, JP24fk0210111, JP24fk0310524, JP24fk0210123, JP24fk0210157, JP24fk0310518, JP24fk0210103, JP24fk0210104, JP24fk0210113, and JP24fk0210143).

## Authors’ contributions

Study design and manuscript revision: RK, GS. Performed examinations and statistical analyses: RK, GS, MO. Sample collection and clinical data: RK, GS, MO, SH, TS, MC, AK, YK, YY, KT, MK, JT, SK, MN, YA, SM, NE, YYamamoto, MB, RY, TSasaki, TY, SY, QF, ZY, OM, SO, YT, TK, NK, MN, MNatsuizaka, KO. Hepatological advice: MNatsuizaka, KO, NS. Revised manuscript for intellectual content: NS.

## Data availability statement

All data generated or analysed during this study are included in this article and the supplementary material. Further inquiries can be directed to the corresponding authors.

## Conflict of interest

NS received lecture fees from Chugai Pharmaceutical Co., and research grants from Gilead Sciences and AbbVie. GS received research grants from Gilead Sciences. AK received a research grant from Chugai Pharmaceutical Co.MK received honoraria for lectures from Eisai Co., Chugai Pharmaceutical Co., Astra Zeneca, Gilead Sciences, and AbbVie. KT received honoraria for lectures from Eisai Co., Chugai Pharmaceutical Co., Astra Zeneca, and Takeda Pharmaceuticals. YYasui received honoraria for lectures from Eisai Co., Chugai Pharmaceutical Co., AstraZeneca, and Eli Lilly. The remaining authors declare no conflicts of interest.

Please refer to the accompanying ICMJE disclosure forms for further details.

## References

[1] Sung H., Ferlay J., Siegel R.L. (2021). Global Cancer Statistics 2020: GLOBOCAN estimates of incidence and mortality worldwide for 36 cancers in 185 countries. CA Cancer J Clin.

[2] Llovet J.M., Kelley R.K., Villanueva A. (2021). Hepatocellular carcinoma. Nat Rev Dis Primers.

[3] Yang X., Wang D., Lin J. (2020). Atezolizumab plus bevacizumab for unresectable hepatocellular carcinoma. Lancet Oncol.

[4] Abou-Alfa G.K., Lau G., Kudo M. (2022). Tremelimumab plus durvalumab in unresectable hepatocellular carcinoma. NEJM Evid.

[5] Lewis J.E., Ebling F.J.P., Samms R.J. (2019). Going back to the biology of FGF21: new insights. Trends Endocrinol Metab.

[6] Liu Z.Y., Luo Y., Fang A.P. (2022). High serum fibroblast growth factor 21 is associated with inferior hepatocellular carcinoma survival: a prospective cohort study. Liver Int.

[7] Hu C., Qiao W., Li X. (2024). Tumor-secreted FGF21 acts as an immune suppressor by rewiring cholesterol metabolism of CD8(+)T cells. Cell Metab.

[8] Suda G., Kudo M., Nagasaka A. (2016). Efficacy and safety of daclatasvir and asunaprevir combination therapy in chronic hemodialysis patients with chronic hepatitis C. J Gastroenterol.

[9] Suda G., Furusyo N., Toyoda H. (2018). Daclatasvir and asunaprevir in hemodialysis patients with hepatitis C virus infection: a nationwide retrospective study in Japan. J Gastroenterol.

[10] Hosoda S., Suda G., Sho T. (2023). Low baseline CXCL9 predicts early progressive disease in unresectable HCC with atezolizumab plus bevacizumab treatment. Liver Cancer.

[11] Sho T., Suda G., Yamamoto Y. (2022). Efficacy and effect on liver functional reserve of atezolizumab and bevacizumab for unresectable hepatocellular carcinoma in patients who do not meet eligibility criteria of IMbrave150. Cancers (Basel).

[12] Sho T., Suda G., Ogawa K. (2021). Early response and safety of atezolizumab plus bevacizumab for unresectable hepatocellular carcinoma in patients who do not meet IMbrave150 eligibility criteria. Hepatol Res.

[13] Suda G., Baba M., Yamamoto Y. (2023). Prophylactic tenofovir alafenamide for hepatitis B virus reactivation and reactivation-related hepatitis. J Med Virol.

[14] Lencioni R., Llovet J.M. (2010). Modified RECIST (mRECIST) assessment for hepatocellular carcinoma. Semin Liver Dis.

[15] Shen Y.C., Liu T.H., Nicholas A. (2024). Clinical outcomes and histologic findings of patients with hepatocellular carcinoma with durable partial response or durable stable disease after receiving atezolizumab plus bevacizumab. J Clin Oncol.

[16] Kohya R., Suda G., Ohara M. (2023). Potential correlation between changes in serum FGF21 levels and lenvatinib-induced appetite loss in patients with unresectable hepatocellular carcinoma. Cancers (Basel).

[17] Finn R.S., Kudo M., Cheng A.L. (2021). Pharmacodynamic biomarkers predictive of survival benefit with lenvatinib in unresectable hepatocellular carcinoma: from the Phase III REFLECT study. Clin Cancer Res.

[18] Yang Z., Suda G., Maehara O. (2023). Changes in serum growth factors during resistance to atezolizumab plus bevacizumab treatment in patients with unresectable hepatocellular carcinoma. Cancers (Basel).

[19] Shigesawa T., Suda G., Kimura M. (2020). Baseline angiopoietin-2 and FGF19 levels predict treatment response in patients receiving multikinase inhibitors for hepatocellular carcinoma. JGH Open.

[20] Yang Z., Suda G., Maehara O. (2022). Changes in serum growth factors during lenvatinib predict the post progressive survival in patients with unresectable hepatocellular carcinoma. Cancers.

[21] Sho T., Suda G., Ogawa K. (2020). Lenvatinib in patients with unresectable hepatocellular carcinoma who do not meet the REFLECT trial eligibility criteria. Hepatol Res.

[22] Finn R.S., Qin S., Ikeda M. (2020). Atezolizumab plus bevacizumab in unresectable hepatocellular carcinoma. N Engl J Med.

[23] Zhu A.X., Abbas A.R., de Galarreta M.R. (2022). Molecular correlates of clinical response and resistance to atezolizumab in combination with bevacizumab in advanced hepatocellular carcinoma. Nat Med.

[24] Shigesawa T., Maehara O., Suda G. (2021). Lenvatinib suppresses cancer stem-like cells in HCC by inhibiting FGFR1-3 signaling, but not FGFR4 signaling. Carcinogenesis.

[25] Qian J., Tikk K., Weigl K. (2018). Fibroblast growth factor 21 as a circulating biomarker at various stages of colorectal carcinogenesis. Br J Cancer.

[26] Li J.R., Chiu K.Y., Ou Y.C. (2019). Alteration in serum concentrations of FGF19, FGF21, and FGF23 in patients with urothelial carcinoma. Biofactors.

[27] Kang Y.E., Kim J.T., Lim M.A. (2019). Association between circulating fibroblast growth factor 21 and aggressiveness in thyroid cancer. Cancers.

[28] Cymbaluk-Ploska A., Gargulinska P., Chudecka-Glaz A. (2020). The suitability of FGF21 and FGF23 as new biomarkers in endometrial cancer patients. Diagnostics (Basel).

[29] Xia J., Zhu Z., Wen G. (2023). Aberrant acetylated modification of FGF21-KLB signaling contributes to hepatocellular carcinoma metastasis through the beta-catenin pathway. Int J Oncol.

[30] Chen J., Jiang S., Shao H. (2022). CRISPR-Cas9-based genome-wide screening identified novel targets for treating sorafenib-resistant hepatocellular carcinoma: a cross-talk between FGF21 and the NRF2 pathway. Sci China Life Sci.

[31] Zheng Q., Martin R.C., Shi X. (2020). Lack of FGF21 promotes NASH-HCC transition via hepatocyte-TLR4-IL-17A signaling. Theranostics.

[32] Xu P., Zhang Y., Wang W. (2015). Long-term administration of fibroblast growth factor 21 prevents chemically-induced hepatocarcinogenesis in mice. Dig Dis Sci.

[33] Harrison S.A., Frias J.P., Neff G. (2023). Safety and efficacy of once-weekly efruxifermin versus placebo in non-alcoholic steatohepatitis (HARMONY): a multicentre, randomised, double-blind, placebo-controlled, phase 2b trial. Lancet Gastroenterol Hepatol.

[34] Zhang I.W., Curto A., Lopez-Vicario C. (2022). Mitochondrial dysfunction governs immunometabolism in leukocytes of patients with acute-on-chronic liver failure. J Hepatol.

[35] Geladari E., Alexopoulos T., Vasilieva L. (2024). Severe underweight and sarcopenia in decompensated cirrhosis are associated with high FGF21 levels. Aliment Pharmacol Ther.

