# Supplementary material for: Serum FGF21 as a predictor of response to atezolizumab and bevacizumab in HCC
Source: JHEP Rep. 2025 Feb 19;7(5):101364. doi: 10.1016/j.jhepr.2025.101364 (PMC11999275; doi:10.1016/j.jhepr.2025.101364)
Supplement: Multimedia component 1 [file mmc1.pdf]

Serum FGF21 as a predictor of response to atezolizumab and bevacizumab in HCC

Risako Kohya, Goki Suda, Masatsugu Ohara, Shunichi Hosoda, Takuya Sho, Makoto Chuma, Atsumasa Komori, Yuki Kugiyama, Yutaka Yasui, Kaoru Tsuchiya, Masayuki Kurosaki, Joji Tani, Shun Kaneko, Mina Nakagawa, Yasuhiro Asahina, Shinya Maekawa, Nobuyuki Enomoto, Yoshiya Yamamoto, Masaru Baba, Ren Yamada, Takashi Sasaki, Tomoka Yoda, Sonoe Yoshida, Qingjie Fu, Zijian Yang, Osamu Maehara, Shunsuke Ohnishi, Yoshimasa Tokuchi, Takashi Kitagataya, Naoki Kawagishi, Masato Nakai, Mitsuteru Natsuizaka, Koji Ogawa, and Naoya Sakamoto

Table of contents

Fig. S1 3
Fig. S2. 4
Fig. S3. 6
Table S1. 8
Table S2. 11
Table S3. 14
Table S4. 17
Table S5 20
Table S6 23

Table S7 26

Table S8 29

Table S9 32

Supplementary Figures

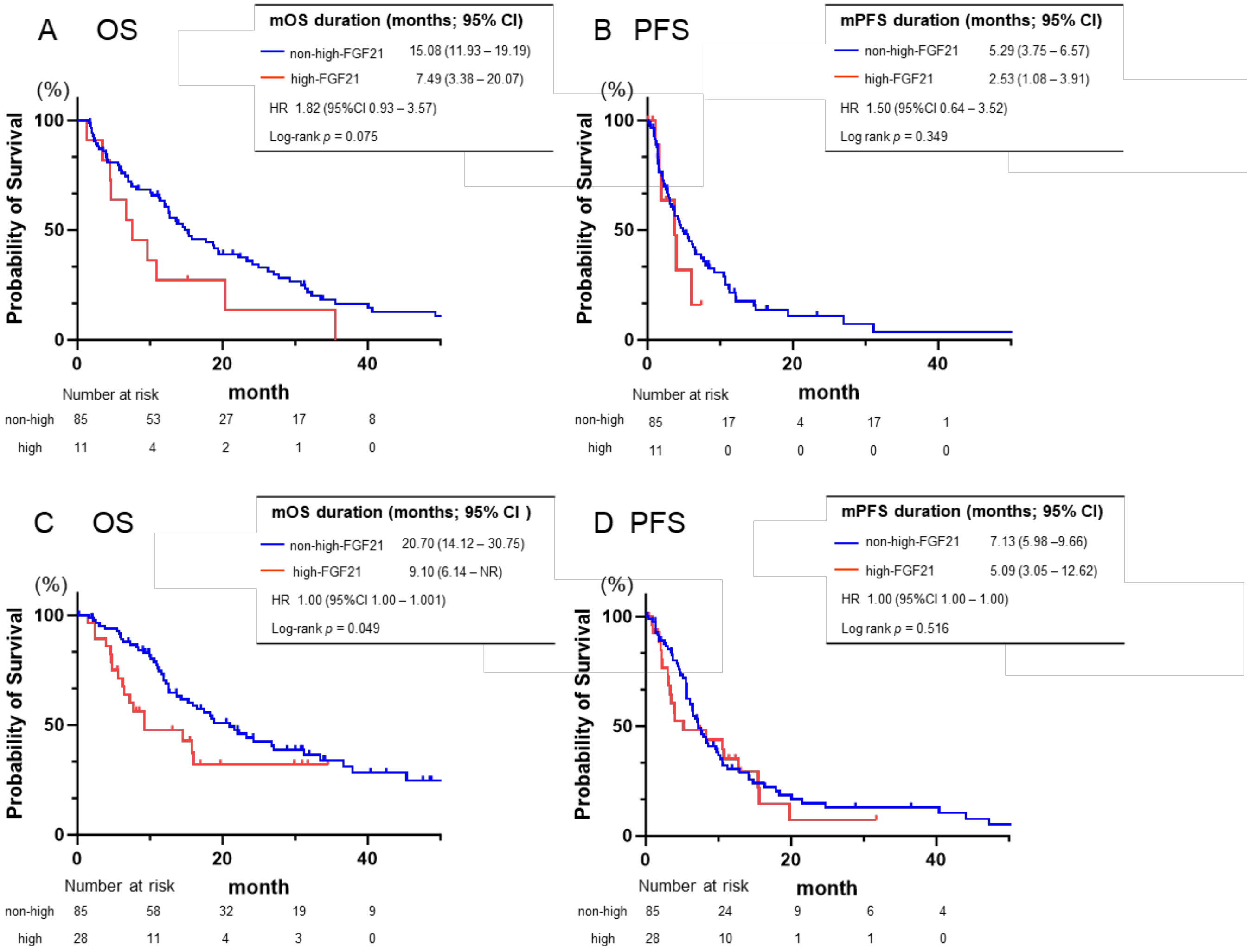

Fig. S1. OS and PFS of patients treated with sorafenib or lenvatinib and stratified according to baseline serum FGF21 levels.

(A, B) Sorafenib. (A) OS; level of significance: p = 0.075 (log-rank test), (B) PFS: level of significance: p = 0.349 (log-rank test). (C, D) Lenvatinib. (C) OS; level of significance: p = 0.049 (log-rank test), (D) PFS: level of significance: p = 0.516 (log-rank test). High-FGF21, high baseline FGF21 levels; non-high-FGF21, non-high baseline FGF21 levels; OS, overall survival; PFS, progression-free survival; HR, hazard ratio; NR, not reached.

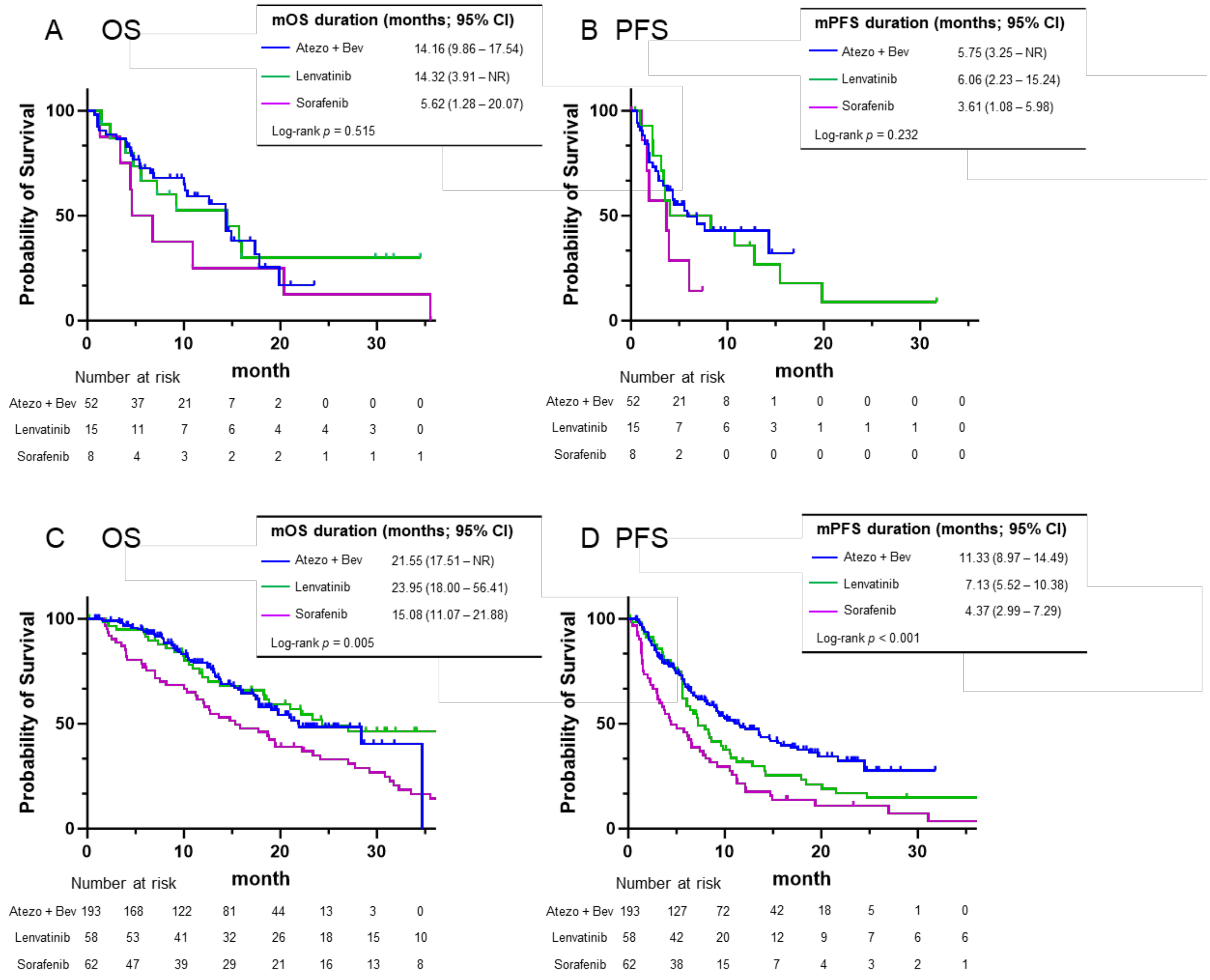

Fig. S2. Comparison of OS and PFS among patients treated with atezolizumab/bevacizumab, lenvatinib, or sorafenib, stratified according to baseline serum FGF21 levels in the first line-limited cohort.

(A, B) Kaplan–Meier estimates of OS and PFS stratified by treatment regimen in patients with high baseline FGF21 levels (high-FGF21). (A) OS; level of significance: $p = 0.515$ (log-rank test). (B) PFS; level of significance: $p = 0.232$ (log-rank test). (C, D) Kaplan–Meier estimates of OS and PFS stratified by treatment regimen in patients with non-high baseline FGF21 levels (non-high-FGF21). (C) OS; level of significance: $p = 0.005$ (log-rank test). (D) PFS; level of

significance: $p < 0.001$ (log-rank test). Atezo/Bev, atezolizumab/bevacizumab; high-FGF21, high baseline FGF21 levels; non-high-FGF21, non-high baseline FGF21 levels; OS, overall survival; PFS, progression-free survival.

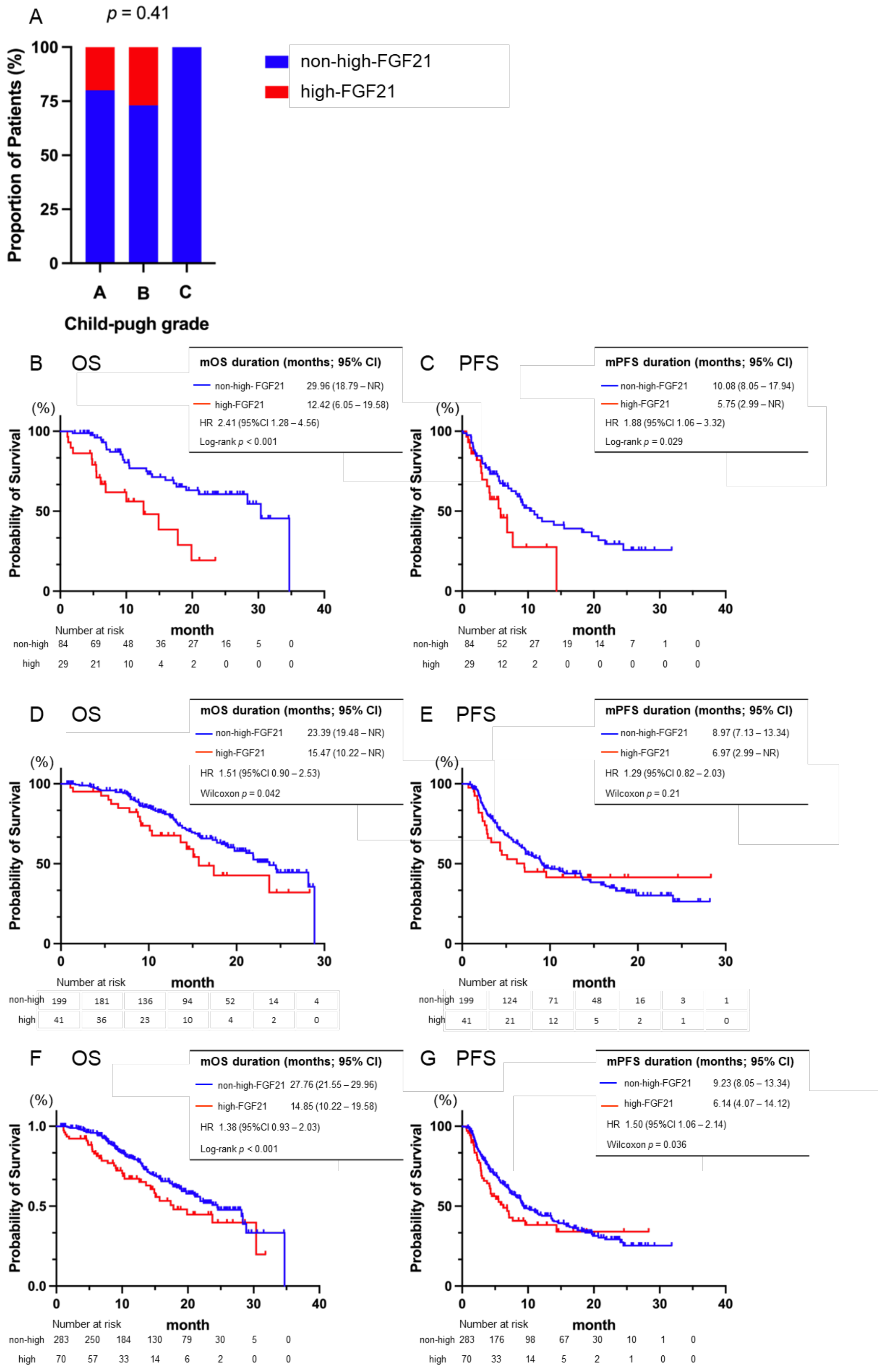

Fig. S3. Relationship between Child–Pugh grade and FGF21 levels, and comparison of OS

and PFS in patients treated with atezolizumab/bevacizumab, stratified by baseline serum FGF21 levels, in a Child–Pugh grade A-restricted cohort.

(A) Fisher's exact test showed no statistically significant association between Child–Pugh grade and baseline serum high FGF21 levels (categorised as high-FGF21 vs. non-high FGF21), with a *p*-value of 0.41. (B, C) Kaplan–Meier estimates of OS and PFS stratified by baseline serum FGF21 levels in patients with Child–Pugh grade A in the discovery cohort. (B) OS; level of significance: $p < 0.001$ (log-rank test). (C) PFS; level of significance: $p = 0.029$ (log-rank test). (D, E) Kaplan–Meier estimates of OS and PFS stratified by baseline serum FGF21 levels in patients with Child–Pugh grade A in the validation cohort. (D) OS; level of significance: $p = 0.042$ (generalised Wilcoxon test). (E) PFS; level of significance: $p = 0.21$ (generalised Wilcoxon test). (F, G) Kaplan–Meier estimates of OS and PFS stratified by baseline serum FGF21 levels in patients with Child–Pugh grade A in the overall cohort. (F) OS; level of significance: $p < 0.001$ (log-rank test). (G) PFS; level of significance: $p = 0.036$ (generalised Wilcoxon test). High-FGF21, high baseline FGF21 levels; non-high-FGF21, non-high baseline FGF21 levels; OS, overall survival; PFS, progression-free survival.

Supplementary Tables

Table S1. Baseline characteristics of patients with non-high and high FGF21 levels in the discovery cohort, categorised using the propensity score.

	Total (n = 52)	Non-high FGF21 (n = 26)	High FGF21 (n = 26)	*P*-value
Age, years	70 (19–86)	69.50 (47–84)	70.50 (19–86)	0.61
Sex, n (%)				
Female	8 (15.4)	3 (11.5)	5 (19.2)	0.70
Male	44 (84.6)	23 (88.5)	21 (80.8)	
BMI, kg/m^2	23.9 (9.80–34.2)	23.95 (19–34.2)	23.8 (9.8–33)	0.41
Aetiology, n (%)				
HBV	16 (30.8)	9 (34.6)	7 (26.9)	0.73
HCV	7 (13.5)	4 (15.4)	3 (11.5)	
NBNC	29 (55.8)	13 (50)	16 (61.5)	
Child–Pugh grade, n (%)				
A	52 (100)	26 (100)	26 (100)	NA
BCLC stage, n (%)				
B	25 (48.1)	13 (50)	12 (46.2)	1
C	27 (51.9)	13 (50)	14 (53.8)	

TNM stage, n (%)				
3	26 (50)	14 (53.8)	12 (46.2)	0.79
4A	6 (11.5)	2 (7.7)	4 (15.4)	
4B	20 (38.5)	10 (38.5)	10 (38.5)	
ALBI grade, n (%)				
1	22 (42.3)	14 (53.8)	8 (30.8)	0.16
2	30 (57.7)	12 (46.2)	18 (69.2)	
FIB-4 index	3.14 (1–15.17)	3.04 (1.62–9.96)	3.50 (1–15.17)	0.57
Best response, n (%)				
PR	13 (25)	6 (23.1)	7 (26.9)	0.70
SD	27 (51.9)	13 (50)	14 (53.8)	
PD	11 (21.2)	7 (26.9)	4 (15.4)	
NA	1 (1.9)	0 (0)	1 (3.8)	
Biochemical analysis				
Plt, $\times 10^4/\mu L$	16.85 (6.1–40.8)	15.95 (8.5–32.0)	18.65 (6.1–40.8)	0.12
PT, %	92.9 (15–142.20)	95.20 (35.3–118.4)	92.1 (15–142.2)	0.97
Alb, g/dL	3.8 (3–4.8)	3.95 (3.2–4.8)	3.65 (3–4.5)	0.04
AST, IU/L	46.5 (14–672)	41.5 (19–106)	48 (14–672)	0.07
ALT, IU/L	30.5 (12–122)	26 (12–122)	32.5 (13–98)	0.42

T-Bil, mg/dL	0.8 (0.4–2.6)	0.8 (0.4–1.6)	0.8 (0.5–2.6)	0.83
AFP, ng/mL	55.2 (0.8–84937.3)	34.1 (0.8–15009.5)	87.9 (2.9–84937.3)	0.28

Data are expressed as median (range) unless otherwise indicated.

Abbreviations: AFP, alpha-fetoprotein; Alb, albumin; ALBI, albumin–bilirubin; ALT, alanine aminotransferase; AST, aspartate aminotransferase; BCLC, Barcelona Clinic Liver Cancer; BMI, body mass index; FIB-4, fibrosis-4 index; HBV, hepatitis B virus; HCV, hepatitis C virus; HR, hazard ratio; NBNC, non-HBV non-HCV; PD, progressive disease; Plt, platelet count; PR, partial response; PT, prothrombin time; SD, stable disease; T-Bil, total bilirubin.

Table S2. Baseline characteristics of patients with non-high and high FGF21 levels in the validation cohort, categorised using the propensity score.

	Total (n = 98)	Non-high FGF21 (n = 49)	High FGF21 (n = 49)	*P*-value
Age, years	76.5 (44–89)	75 (44–89)	78 (53–89)	0.77
Sex, n (%)				
Female	20 (20.4)	9 (18.4)	11 (22.4)	0.8
Male	78 (79.6)	40 (81.6)	38 (77.6)	
BMI, kg/m^2	23.1 (14.38–36.64)	22.8 (15–34.23)	23.43 (14.38–36.64)	0.66
Aetiology, n (%)				
HBV	16 (16.3)	8 (16.3)	8 (16.3)	0.86
HCV	33 (33.7)	18 (36.7)	15 (30.6)	
NBNC	49 (50)	23 (46.9)	26 (53.1)	
Child–Pugh grade, n (%)				
A	75 (76.5)	37 (75.5)	38 (77.6)	1
B	23 (23.5)	12 (24.5)	11 (22.4)	
TNM stage, n (%)				
2	17 (17.3)	9 (18.4)	8 (16.3)	0.89
3	30 (30.6)	14 (28.6)	16 (32.7)	

4A	5 (5.1)	2 (4.1)	3 (6.1)	
4B	14 (14.3)	6 (12.2)	8 (16.3)	
BCLC stage, n (%)				
B	37 (37.8)	18 (36.7)	19 (38.8)	1
C	61 (62.2)	31 (63.3)	30 (61.2)	
ALBI grade, n (%)				
1	25 (25.5)	17 (34.7)	8 (16.3)	0.09
2	70 (71.4)	31 (63.3)	39 (79.6)	
3	3 (3.1)	1 (2)	2 (4.1)	
FIB-4 index	3.66 (0.12–15.75)	2.78 (0.17–15.35)	4.14 (0.12–15.75)	0.18
Best response, n (%)				
CR	3 (3.1)	3 (6.1)	0 (0)	0.02
PR	27 (27.6)	11 (22.4)	16 (32.7)	
SD	29 (29.6)	20 (40.8)	9 (18.4)	
PD	22 (22.4)	7 (14.3)	15 (30.6)	
NA	17 (17.3)	8 (16.3)	9 (18.4)	
Biochemical analysis				
Plt, $\times 10^4/\mu L$	14.4 (3.7–40.3)	14.3 (3.7–29.1)	14.5 (8.40–40.3)	0.21
PT, %	92 (41–131)	92 (49–131)	92.3 (41–131)	0.61

Alb, g/dL	3.6 (2.4–4.7)	3.6 (2.5–4.7)	3.4 (2.4–4.5)	0.01
AST, IU/L	45.50 (15–260)	38 (16–259)	53 (15–260)	0.03
ALT, IU/L	28.5 (8–147)	31 (8–127)	27 (9–147)	0.78
T-Bil, mg/dL	0.8 (0.3–3)	0.8 (0.3–2)	0.8 (0.4–3)	0.54
AFP, ng/mL	82.8 (0.5–1133450)	41.8 (2–1133450)	82.8 (0.5–628992)	0.27

Data are expressed as median (range) unless otherwise indicated.

Abbreviations: AFP, alpha-fetoprotein; Alb, albumin; ALBI, albumin–bilirubin; ALT, alanine aminotransferase; AST, aspartate aminotransferase; BCLC, Barcelona Clinic Liver Cancer; BMI, body mass index; CR, complete response; FIB-4, fibrosis-4 index; HBV, hepatitis B virus; HCV, hepatitis C virus; HR, hazard ratio; NBNC, non-HBV non-HCV; PD, progressive disease; Plt, platelet count; PR, partial response; PT, prothrombin time; SD, stable disease; T-Bil, total bilirubin.

Table S3. Baseline characteristics of patients with non-high and high FGF21 levels in the overall cohort, categorised using the propensity score.

	Total (n = 156)	Non-high FGF21 (n = 78)	High FGF21 (n = 78)	*P*-value
Age, years	43 (27.6)	26 (33.3)	17 (21.8)	0.28
Sex, n (%)				
Female	36 (23.1)	19 (24.4)	17 (21.8)	0.85
Male	120 (76.9)	59 (75.6)	61 (78.2)	
BMI, kg/m^2	23.4 (9.8–36.64)	22.99 (15.02–33.96)	23.8 (9.8–36.64)	0.71
Aetiology, n (%)				
HBV	32 (20.5)	16 (20.5)	16 (20.5)	0.04
HCV	52 (33.3)	33 (42.3)	19 (24.4)	
NBNC	72 (46.2)	29 (37.2)	43 (55.1)	
TNM stage, n (%)				
2	16 (10.3)	7 (9)	9 (11.5)	0.96
3	59 (37.8)	31 (39.7)	28 (35.9)	
4A	5 (3.2)	2 (2.6)	3 (3.8)	
4B	23 (14.7)	12 (15.4)	11 (14.1)	
Child–Pugh grade, n (%)				

A	137 (87.8)	71 (91)	66 (84.6)	0.33
B	19 (12.2)	7 (9)	12 (15.4)	
BCLC stage, n (%)				
B	63 (40.4)	32 (41)	31 (39.7)	1
C	93 (59.6)	46 (59)	47 (60.3)	
ALBI grade, n (%)				
1	43 (27.6)	26 (33.3)	17 (21.8)	0.28
2	109 (69.9)	50 (64.1)	59 (75.6)	
3	4 (2.6)	2 (2.6)	2 (2.6)	
FIB-4 index	3.54 (0.12–32.8)	3.25 (0.12–32.8)	3.84 (0.12–15.75)	0.26
Best response, n (%)				
CR	5 (3.2)	5 (6.4)	0 (0)	0.06
PR	43 (27.6)	20 (25.6)	23 (29.5)	
SD	59 (37.8)	33 (42.3)	26 (33.3)	
PD	29 (18.6)	10 (12.8)	19 (24.4)	
NA	20 (12.8)	10 (12.8)	10 (12.8)	
Biochemical analysis				
Plt, ×104/μL	15.45 (5.4–44.9)	14.25 (5.4–44.9)	16.5 (6.1–41.9)	0.02
PT, %	92.15 (15–146.1)	92.1 (50.7–146.1)	92.15 (15–142.2)	0.86

Alb, g/dL	3.6 (2.1–4.8)	3.60 (2.1–4.8)	3.5 (2.4–4.5)	0.07
AST, IU/L	47 (12–672)	43 (12–272)	52.50 (14–672)	0.04
ALT, IU/L	29 (6–127)	29 (6–127)	28.50 (9–113)	0.99
T-Bil, mg/dL	0.8 (0.3–3)	0.8 (0.3–2.4)	0.8 (0.4–3)	0.26
AFP, ng/mL	105.3 (0.5–852122.7)	107.8 (1.3–852122.7)	98.55 (0.5–628992)	0.31

Data are expressed as median (range) unless otherwise indicated.

Abbreviations: AFP, alpha-fetoprotein; Alb, albumin; ALBI, albumin–bilirubin; ALT, alanine aminotransferase; AST, aspartate aminotransferase; BCLC, Barcelona Clinic Liver Cancer; BMI, body mass index; CR, complete response; FIB-4, fibrosis-4 index; HBV, hepatitis B virus; HCV, hepatitis C virus; HR, hazard ratio; NBNC, non-HBV non-HCV; PD, progressive disease; Plt, platelet count; PR, partial response; PT, prothrombin time; SD, stable disease; T-Bil, total bilirubin.

Table S4. Baseline characteristics of patients with non-high and high FGF21 levels in the sorafenib treatment cohort.

	Total (n = 96)	Non-high FGF21 (n = 85)	High FGF21 (n = 11)	*P*-value
Age, years	69 (34–90)	69 (34–90)	70 (48–88)	0.96
Sex, n (%)				
Female	17 (17.7)	14 (16.5)	3 (27.3)	0.41
Male	79 (82.3)	71 (83.5)	8 (72.7)	
BMI, kg/m^2	23.41 (14.89–38.89)	23.52 (14.89–38.89)	22.27 (19.19–36.7)	0.33
Aetiology, n (%)				
HBV	36 (37.5)	32 (37.6)	4 (36.4)	0.63
HCV	22 (22.9)	21 (24.7)	1 (9.1)	
HBV/HCV	2 (2.1)	2 (2.4)	0 (0)	
HCV+ALC	1 (1)	1 (1.2)	0 (0)	
NBNC	34 (35.4)	28 (32.9)	6 (54.5)	
NA	1 (1)	1 (1.2)	0 (0)	
Child–Pugh grade, n (%)				
A	74 (77.1)	67 (78.8)	7 (63.6)	0.27
B	22 (22.9)	18 (21.2)	4 (36.4)	

BCLC stage, n (%)				
B	47 (49)	42 (49.4)	5 (45.5)	1
C	49 (51)	43 (50.6)	6 (54.5)	
ALBI grade, n (%)				
1	24 (25)	23 (27.1)	1 (9.1)	0.13
2	67 (69.8)	59 (69.4)	8 (72.7)	
3	5 (5.2)	3 (3.5)	2 (18.2)	
FIB-4 index	4.76 (0.83–24.92)	4.78 (0.83–24.92)	4.74 (1.14–20.21)	0.94
Best response, n (%)				
CR	3 (4.1)	3 (4.6)	0 (0)	0.67
PR	11 (14.9)	11 (16.9)	0 (0)	
SD	32 (43.2)	27 (41.5)	5 (55.6)	
PD	28 (37.8)	24 (36.9)	4 (44.4)	
Biochemical analysis				
Plt, $\times 10^4/\mu L$	11.45 (4.3–48)	11.5 (4.3–48)	1.6 (5.4–34.5)	0.87
PT, %	85.45 (44.3–129.4)	85.4 (44.3–129.4)	89 (56–11.3)	0.98
Alb, g/dL	3.5 (1.8–4.6)	3.5 (2.5–4.6)	3.3 (1.8–3.9)	0.17
AST, IU/L	42.5 (13–247)	43 (15–186)	37 (13–247)	0.93

ALT, IU/L	33 (7–157)	33 (7–157)	39 (9–128)	0.49
T-Bil, mg/dL	0.9 (0.2–9)	0.9 (0.2–9)	1.4 (0.5–2.2)	0.101
AFP, ng/mL	75.1 (1–193374)	66 (1–193374)	33.2 (3–61558)	0.42

Data are expressed as median (range) unless otherwise indicated.

Abbreviations: AFP, alpha-fetoprotein; Alb, albumin; ALBI, albumin–bilirubin; ALT, alanine aminotransferase; AST, aspartate aminotransferase; BCLC, Barcelona Clinic Liver Cancer; BMI, body mass index; CR, complete response; FIB-4, fibrosis-4 index; HBV, hepatitis B virus; HCV, hepatitis C virus; HR, hazard ratio; NBNC, non-HBV non-HCV; PD, progressive disease; Plt, platelet count; PR, partial response; PT, prothrombin time; SD, stable disease; T-Bil, total bilirubin.

Table S5. Baseline characteristics of patients with non-high and high FGF21 levels in the lenvatinib treatment cohort.

	Total (n = 113)	Non-high FGF21 (n = 85)	High FGF21 (n = 28)	*P*-value
Age, years	70 (31–88)	69. (31–84)	74 (47–88)	0.02
Sex, n (%)				
Female	15 (13.3)	10 (11.8)	5 (17.9)	0.52
Male	98 (86.7)	75 (88.2)	23 (82.1)	
BMI, kg/m^2	23.8 (17.04–48.9)	23.67 (17.04–35.36)	24.17 (17.57–48.9)	0.4
Aetiology, n (%)				
HBV	34 (30.1)	28 (32.9)	6 (21.4)	0.53
HCV	18 (15.9)	13 (15.3)	5 (17.9)	
NBNC	61 (54)	44 (51.8)	17 (60.7)	
Child–Pugh grade, n (%)				
A	82 (72.6)	64 (75.3)	18 (64.3)	0.33
B	31 (27.4)	21 (24.7)	10 (35.7)	
BCLC stage, n (%)				
A	1 (0.9)	0 (0)	1 (3.6)	0.11
B	52 (46.0)	42 (49.4)	10 (35.7)	

C	60 (53.1)	43 (50.6)	17 (60.7)	
ALBI grade, n (%)				
1	34 (30.1)	30 (35.3)	4 (14.3)	0.03
2	72 (63.7)	52 (61.2)	20 (71.4)	
3	7 (6.2)	3 (3.5)	4 (14.3)	
FIB-4 index	3.8 (0.7–22.42)	3.7 (0.7–22.42)	4.94 (0.81–14.73)	0.17
Best response, n (%)				
CR	16 (14.4)	12 (14.3)	4 (14.8)	0.51
PR	37 (33.3)	29 (34.5)	8 (29.6)	
SD	46 (41.4)	36 (42.9)	10 (37)	
PD	12 (10.8)	7 (8.3)	5 (18.5)	
Biochemical analysis				
Plt, $\times 10^4/\mu L$	15.7 (4.2–51.7)	13.6 (4.4–50)	17 (4.2–51.7)	0.061
PT, %	86.8 (12.1–127.9)	85.9 (12.1–127.9)	92.5 (23.7–117.7)	0.398
Alb, g/dL	3.60 (2.3–4.6)	3.7 (2.5–4.6)	3.15 (2.3–4.1)	0.001
AST, IU/L	38 (15–303)	37 (15–303)	68 (18–220)	0.001
ALT, IU/L	26 (6–168)	24 (6–94)	33 (13–168)	0.1
T-Bil, mg/dL	0.9 (0.3–4)	0.9 (0.3–4)	0.7 (0.3–2.1)	0.09
AFP, ng/mL	39.4 (1.3–449909)	32 (1.3–449909)	162 (1.6–290835)	0.124

Data are expressed as median (range) unless otherwise indicated.

Abbreviations: AFP, alpha-fetoprotein; Alb, albumin; ALBI, albumin–bilirubin; ALT, alanine aminotransferase; AST, aspartate aminotransferase; BCLC, Barcelona Clinic Liver Cancer; BMI, body mass index; CR, complete response; FIB-4, fibrosis-4 index; HBV, hepatitis B virus; HCV, hepatitis C virus; HR, hazard ratio; NBNC, non-HBV non-HCV; PD, progressive disease; Plt, platelet count; PR, partial response; PT, prothrombin time; SD, stable disease; T-Bil, total bilirubin.

Table S6. Baseline characteristics **of** patients with high baseline FGF21 levels **treated with sorafenib, lenvatinib, or atezolizumab/bevacizumab.**

	Atezo/Bev (n = 83)	Lenvatinib (n = 28)	Sorafenib (n = 11)	*P*-value
Age (years)	74 (19–89)	74 (47–88)	70 (48–88)	0.33
Sex (%)				
Female	18 (21.7)	5 (17.9)	3 (27.3)	0.78
Male	65 (78.3)	23 (82.1)	8 (72.7)	
BMI (kg/m^2)	23.43 (9.8–36.64)	24.17 (17.57–48.9)	22.27 (19.19–36.7)	0.25
Aetiology, n (%)				
HBV	19 (22.9)	6 (21.4)	4 (36.4)	0.77
HCV	19 (22.9)	5 (17.9)	1 (9.1)	
NBNC	45 (54.2)	17 (60.7)	6 (54.5)	
Child–Pugh grade n (%)				
A	70 (84.3)	18 (64.3)	7 (63.6)	0.04
B	13 (15.7)	10 (35.7)	4 (36.4)	
BCLC stage, n (%)				
A	0 (0)	1 (3.6)	0 (0)	0.5
B	32 (38.6)	10 (35.7)	5 (45.5)	

C	51 (61.4)	17 (60.7)	6 (54.5)	
ALBI grade (%)				
1	17 (20.5)	4 (14.3)	1 (9.1)	0.07
2	64 (77.1)	20 (71.4)	8 (72.7)	
3	2 (2.4)	4 (14.3)	2 (18.2)	
FIB-4 index	3.83 (0.12–15.75)	4.94 (0.81–14.73)	4.74 (1.14–20.21)	0.35
Best overall response, n (%)				
CR	0 (0)	4 (14.3)	0 (0)	0.01
PR	23 (31.9)	8 (28.6)	0 (0)	
SD	27 (37.5)	10 (35.7)	5 (45.5)	
PD	22 (30.6)	5 (17.9)	4 (36.4)	
NA	11 (13.3)	1 (3.6)	2 (18.2)	
Biochemical analysis				
Plt, $\times 10^4/\mu L$	16.80 (6.1–41.9)	17 (4.2–51.7)	10.6 (5.4–34.5)	0.37
PT, %	92 (15–142.2)	92.5 (23.7–117.7)	89 (56–101.3)	0.46
Alb, g/dL	3.5 (2.4–4.5)	3.2 (2.3–4.1)	3.3 (1.8–3.9)	0.11
AST, IU/L	54 (14–672)	68 (18–220)	37 (13–247)	0.66
ALT, IU/L	30 (9–386)	33 (13–168)	39 (9–128)	0.93
T-Bil, mg/dl	0.8 (0.4–3)	0.7 (0.3–2.1)	1.4 (0.5–2.2)	0.05

AFP, ng/mL	82.8 (0.5–628992)	162 (1.6–290835)	330.2 (3–61558)	0.84

Data are expressed as median (range) unless otherwise indicated.

Abbreviations: AFP, alpha-fetoprotein; Alb, albumin; ALBI, albumin–bilirubin; ALT, alanine aminotransferase; AST, aspartate aminotransferase; BCLC, Barcelona Clinic Liver Cancer; BMI, body mass index; CR, complete response; FIB-4, fibrosis-4 index; HBV, hepatitis B virus; HCV, hepatitis C virus; HR, hazard ratio; NBNC, non-HBV non-HCV; PD, progressive disease; Plt, platelet count; PR, partial response; PT, prothrombin time; SD, stable disease; T-Bil, total bilirubin.

Table S7. Baseline characteristics **of** patients with high baseline FGF21 levels **treated with sorafenib, lenvatinib, or atezolizumab/bevacizumab in the first line-limited cohort.**

	Atezo/Bev (n = 52)	Lenvatinib (n = 15)	Sorafenib (n = 8)	*P*-value
Age (years)	74 (46–89)	77 (50–88)	60 (48–88)	0.22
Sex (%)				
Female	11 (21.2)	5 (33.3)	2 (25.0)	0.58
Male	41 (78.8)	10 (66.7)	6 (75.0)	
BMI (kg/m^2)	23.55 (9.8–33)	24.2 (17.57–48.9)	21.37 (19.19–36.7)	0.51
Aetiology, n (%)				
HBV	14 (26.9)	3 (20)	4 (50)	0.72
HCV	10 (19.2)	3 (20)	1 (12.5)	
NBNC	28 (53.8)	9 (60)	3 (37.5)	
Child–Pugh grade n (%)				
A	43 (82.7)	9 (60)	4 (50)	0.04
B	9 (17.3)	6 (40)	4 (50)	
BCLC stage, n (%)				
A	0 (0)	1 (6.7)	0 (0)	0.23
B	18 (34.6)	3 (20)	4 (50)	

C	34 (65.4)	11 (73.3)	4 (50)	
ALBI grade (%)				
1	9 (17.3)	3 (20)	0 (0)	0.03
2	43 (82.7)	11 (73.3)	6 (75)	
3	0 (0)	1 (6.7)	2 (25)	
FIB-4 index	3.71 (0.12–15.75)	5.78 (0.81–14.13)	5.46 (1.14–20.21)	0.47
Best overall response, n (%)				
NA	7 (13.5)	1 (6.7)	2 (25.0)	0.02
CR	0 (0.0)	3 (20.0)	0 (0.0)	
PR	13 (25.0)	1 (6.7)	0 (0.0)	
SD	19 (36.5)	7 (46.7)	3 (37.5)	
PD	13 (25.0)	3 (20.0)	3 (37.5)	
Biochemical analysis				
Plt, $\times 10^4/\mu L$	19.2 (8.4–41.9)	19.3 (7.6–51.7)	8.45 (5.4–34.5)	0.22
PT, %	89.8 (15–142.2)	97.3 (23.7–117.7)	87.65 (56–101.3)	0.68
Alb, g/dL	3.5 (2.4–4.5)	3.2 (2.5–4.1)	3.25 (1.8–3.8)	0.10
AST, IU/L	54 (14–672)	51 (18–220)	38.5 (25–247)	0.73
ALT, IU/L	30.5 (9–386)	30 (13–168)	49 (15–128)	0.65
T-Bil, mg/dl	0.9 (0.4–3)	0.7 (0.3–1.5)	1.5 (0.7–2.2)	0.01

AFP, ng/mL	62.75 (2–628992)	210.9 (1.6–290835)	514.55 (6.4–61558)	0.61

Data are expressed as median (range) unless otherwise indicated.

Abbreviations: AFP, alpha-fetoprotein; Alb, albumin; ALBI, albumin–bilirubin; ALT, alanine aminotransferase; AST, aspartate aminotransferase; BCLC, Barcelona Clinic Liver Cancer; BMI, body mass index; CR, complete response; FIB-4, fibrosis-4 index; HBV, hepatitis B virus; HCV, hepatitis C virus; HR, hazard ratio; NBNC, non-HBV non-HCV; PD, progressive disease; Plt, platelet count; PR, partial response; PT, prothrombin time; SD, stable disease; T-Bil, total bilirubin.

Table S8. Baseline characteristics **of** patients with non-high baseline FGF21 levels **treated with sorafenib, lenvatinib, or atezolizumab/bevacizumab.**

	Atezo/Bev (n = 319)	Lenvatinib (n = 85)	Sorafenib (n = 85)	*P*-value
Age (years)	73 (24–91)	69 (31–84)	69 (34–90)	<0.001
Sex (%)				
Female	63 (19.7)	10 (11.8)	14 (16.5)	0.23
Male	256 (80.3)	75 (88.2)	71 (83.5)	
BMI (kg/m^2)	23.40 (15–41.62)	23.67 (17.04–35.36)	23.52 (14.89–38.89)	0.39
Aetiology, n (%)				
HBV	67 (21)	28 (32.9)	32 (37.6)	NA
HBV/HCV	0 (0)	0 (0)	2 (2.4)	
HCV	101 (31.7)	13 (15.3)	21 (24.7)	
HCV+ALC	0 (0)	0 (0)	1 (1.2)	
NBNC	151 (47.3)	44 (51.8)	28 (32.9)	
NA	0 (0)	0 (0)	1 (1.2)	
Child–Pugh grade n (%)				
A	283 (88.7)	64 (75.3)	67 (78.8)	0.004
B	35 (11)	21 (24.7)	18 (21.2)	

C	1 (0.3)	0 (0)	0 (0)	
BCLC stage, n (%)				
A	8 (2.5)	0 (0)	0 (0)	0.49
B	147 (46.1)	42 (49.4)	42 (49.4)	
C	164 (51.4)	43 (50.6)	43 (50.6)	
ALBI grade (%)				
1	97 (30.4)	30 (35.3)	23 (27.1)	NA
2	213 (66.8)	52 (61.2)	59 (69.4)	
3	9 (2.8)	3 (3.5)	3 (3.5)	
FIB-4 index	2.93 (0.09–32.8)	3.7 (0.7–22.42)	4.78 (0.83–24.92)	<0.001
Best overall response, n (%)				
CR	13 (4.1)	12 (14.1)	3 (3.5)	<0.001
PR	84 (26.3)	29 (34.1)	11 (12.9)	
SD	127 (39.8)	36 (42.4)	27 (31.8)	
PD	62 (19.4)	7 (8.2)	24 (28.2)	
NA	33 (10.3)	1 (1.2)	20 (23.5)	
Biochemical analysis				
Plt, × 10^4/μL	14.3 (3.6–46.4)	13.6 (4.4–50)	11.5 (4.3–48)	0.02
PT, %	92 (34–155.3)	85.9 (12.1–127.9)	85.4 (44.3–129.4)	<0.001

Alb, g/dL	3.7 (2.6–4.8)	3.8 (2.7–4.6)	3.6 (2.5–4.6)	0.66
AST, IU/L	38 (3–349)	37 (15–303)	43 (15–186)	0.054
ALT, IU/L	28 (5–174)	24 (6–94)	33 (7–157)	0.08
T-Bil, mg/dl	0.8 (0.3–2.9)	0.9 (0.3–4)	0.9 (0.2–9)	0.16
AFP, ng/mL	80.1 (0.8–1133450)	32 (1.3–449909)	66 (1–193374)	0.18

Data are expressed as median (range) unless otherwise indicated.

Abbreviations: AFP, alpha-fetoprotein; Alb, albumin; ALBI, albumin–bilirubin; ALT, alanine aminotransferase; AST, aspartate aminotransferase; BCLC, Barcelona Clinic Liver Cancer; BMI, body mass index; CR, complete response; FIB-4, fibrosis-4 index; HBV, hepatitis B virus; HCV, hepatitis C virus; HR, hazard ratio; NBNC, non-HBV non-HCV; PD, progressive disease; Plt, platelet count; PR, partial response; PT, prothrombin time; SD, stable disease; T-Bil, total bilirubin.

Table S9. Baseline characteristics **of** patients with non-high baseline FGF21 levels **treated with sorafenib, lenvatinib, or atezolizumab/bevacizumab in the first line-limited cohort.**

	Atezo/Bev (n = 193)	Lenvatinib (n = 58)	Sorafenib n = 62	*P*-value
Age (years)	73 (31–90)	70 (31–84)	66 (34–90)	<0.001
Sex (%)				
Female	43 (22.3)	4 (6.9)	11 (17.7)	0.021
Male	150 (77.7)	54 (93.1)	51 (82.3)	
BMI (kg/m^2)	23.60 (15–35)	23.60 (17.32–34.10)	23.73 (14.89–38.89)	0.7
Aetiology, n (%)				
HBV	41 (21.2)	15 (25.9)	28 (45.2)	0.001
HCV	65 (33.7)	10 (17.2)	17 (27.4)	
NBNC	87 (45.1)	33 (56.9)	17 (27.4)	
Child–Pugh grade n (%)				
A	173 (89.6)	44 (75.9)	49 (79)	0.011
B	20 (10.4)	14 (24.1)	13 (21)	
BCLC stage, n (%)				
A	6 (3.1)	0 (0)	0 (0)	0.56
B	91 (47.2)	31 (53.4)	31 (50)	

C	96 (49.7)	27 (46.6)	31 (50)	
ALBI grade (%)				
1	69 (35.8)	20 (34.5)	20 (32.3)	0.83
2	119 (61.7)	35 (60.3)	40 (64.5)	
3	5 (2.6)	3 (5.2)	2 (3.2)	
FIB-4 index	2.82 (0.09–32.80)	3.50 (0.70–22.42)	4.72 (1.05–18.61)	<0.001
Best overall response, n (%)				
CR	8 (4.1)	9 (15.3)	2 (3.2)	<0.001
PR	58 (30.1)	21 (35.6)	7 (11.3)	
SD	26 (13.5)	23 (39.0)	18 (29.0)	
PD	81 (42.0)	5 (8.5)	18 (29.0)	
NA	20 (10.4)	1 (1.7)	17 (27.4)	
Biochemical analysis				
Plt, $\times 10^4/\mu L$	14.30 (3.7–46.4)	13.75 (5.1–50)	11 (4.5–43.2)	0.004
PT, %	89.8 (34–155.3)	85.25 (12.1–127.9)	81.55 (44.3–124)	0.003
Alb, g/dL	3.8 (2.1–4.8)	3.75 (2.5–4.6)	3.6 (2.7–4.6)	0.64
AST, IU/L	38 (11–259)	37 (15–303)	42.50 (20–186)	0.13
ALT, IU/L	27 (6–127)	25 (6–94)	32 (8–157)	0.24
T-Bil, mg/dl	0.8 (0.3–2.9)	0.9 (0.4–2.6)	0.9 (0.3–9)	0.099

AFP, ng/mL	28.9 (1.2–1133450)	14.9 (1.3–254560.8)	67.60 (1–193374)	0.067

Data are expressed as median (range) unless otherwise indicated.

Abbreviations: AFP, alpha-fetoprotein; Alb, albumin; ALBI, albumin–bilirubin; ALT, alanine aminotransferase; AST, aspartate aminotransferase; BCLC, Barcelona Clinic Liver Cancer; BMI, body mass index; CR, complete response; FIB-4, fibrosis-4 index; HBV, hepatitis B virus; HCV, hepatitis C virus; HR, hazard ratio; NBNC, non-HBV non-HCV; PD, progressive disease; Plt, platelet count; PR, partial response; PT, prothrombin time; SD, stable disease; T-Bil, total bilirubin.
